# A design strategy for high mobility stretchable polymer semiconductors

**DOI:** 10.1038/s41467-021-23798-2

**Published:** 2021-06-11

**Authors:** Jaewan Mun, Yuto Ochiai, Weichen Wang, Yu Zheng, Yu-Qing Zheng, Hung-Chin Wu, Naoji Matsuhisa, Tomoya Higashihara, Jeffrey B.-H. Tok, Youngjun Yun, Zhenan Bao

**Affiliations:** 1 Department of Chemical Engineering, Stanford University, Stanford, CA USA; 2 Department of Organic Materials Science, Yamagata University, Yonezawa, Yamagata Japan; 3 Department of Materials Science and Engineering, Stanford University, Stanford, CA USA; 4 Department of Chemistry, Stanford University, Stanford, CA USA; 5 Samsung Advanced Institute of Technology (SAIT), Samsung Electronics, Suwon, South Korea; 6 Present Address: Department of Electronics and Electrical Engineering, Keio University, Kohoku-ku, Yokohama Japan

**Keywords:** Electronic devices, Polymers

## Abstract

As a key component in stretchable electronics, semiconducting polymers have been widely studied. However, it remains challenging to achieve stretchable semiconducting polymers with high mobility and mechanical reversibility against repeated mechanical stress. Here, we report a simple and universal strategy to realize intrinsically stretchable semiconducting polymers with controlled multi-scale ordering to address this challenge. Specifically, incorporating two types of randomly distributed co-monomer units reduces overall crystallinity and longer-range orders while maintaining short-range ordered aggregates. The resulting polymers maintain high mobility while having much improved stretchability and mechanical reversibility compared with the regular polymer structure with only one type of co-monomer units. Interestingly, the crystalline microstructures are mostly retained even under strain, which may contribute to the improved robustness of our stretchable semiconductors. The proposed molecular design concept is observed to improve the mechanical properties of various p- and n-type conjugated polymers, thus showing the general applicability of our approach. Finally, fully stretchable transistors fabricated with our newly designed stretchable semiconductors exhibit the highest and most stable mobility retention capability under repeated strains of 1,000 cycles. Our general molecular engineering strategy offers a rapid way to develop high mobility stretchable semiconducting polymers.

## Abstract (web summary)

Designing intrinsically stretchable semiconducting polymers with suitable charge transport and mechanical properties required for stretchable electronic devices remains a challenge. Here, the authors report terpolymer-based semiconductors with intrinsically high stretchability and mobility.

## Introduction

With increasing needs for wearable and implantable electronics, flexible and stretchable electronics have developed recently^1–9^. Such electronics with new form factors will undoubtedly improve our daily life in the future^10^. A number of promising applications, such as stretchable circuits^11–16^, displays^17–22^, and energy storage devices^23–27^, have been demonstrated. As a key component in electronics, semiconductors that are intrinsically stretchable will allow the fabrication of high-density devices and create more robust products. Stretchable semiconducting polymers have several additional advantages such as low-cost solution processability and scalability^28,29^; however, several challenges remain to be overcome for stretchable semiconducting polymers. For example, charge carrier mobility is often reduced with increasing stretchability, which significantly limits the use of stretchable semiconducting polymers^30,31^. Even though significant progress has been made with the incorporation of dynamic bonding units, partial breakage of the conjugation makes it challenging to control the morphology, which makes it difficult to realize high mobility^32–34^. Several types of additives have been reported and have shown some promise. However, such multi-component systems are highly sensitive to processing conditions^35–37^. In some approaches, specific molecular combinations are required to achieve high stretchability^38^, which may limit the broad application of such methods. Therefore, it is desired to develop a molecular design strategy that is easy to access, reliable without phase-separation, generally applicable to a variety of known high-mobility polymer semiconductors, and offers high stretchability without compromising charge carrier mobility.

Herein, we report a simple and general molecular design strategy for high-mobility intrinsically stretchable polymer semiconductors to address the above limitations. Specifically, stretchable terpolymer-based (comprising three discrete conjugated building blocks) semiconductors were synthesized by utilizing various fractions of two types of constituting co-monomers. The afforded semiconducting terpolymers are found to be stretchable to >100% strain without crack formation. As no additives are needed to tune the mechanical properties, these polymers readily give high mobilities and high stretchability. The molecular design concept is found to be generally applicable and thus allows the rapid development of a variety of stretchable *p*-type and *n*-type semiconductors. We hypothesized that the introduction of two different types of fully conjugated co-monomers may not significantly affect the short-range aggregation of polymeric chains, whereas the structural randomness of the backbone may hinder the formation of larger crystalline domains, which tend to fracture upon strain^39^. This controlled ordering at different length scales may improve stretchability without compromise of charge transport.

## Results

### Improved mechanical properties without compromise in mobility

**Fig. 1:**
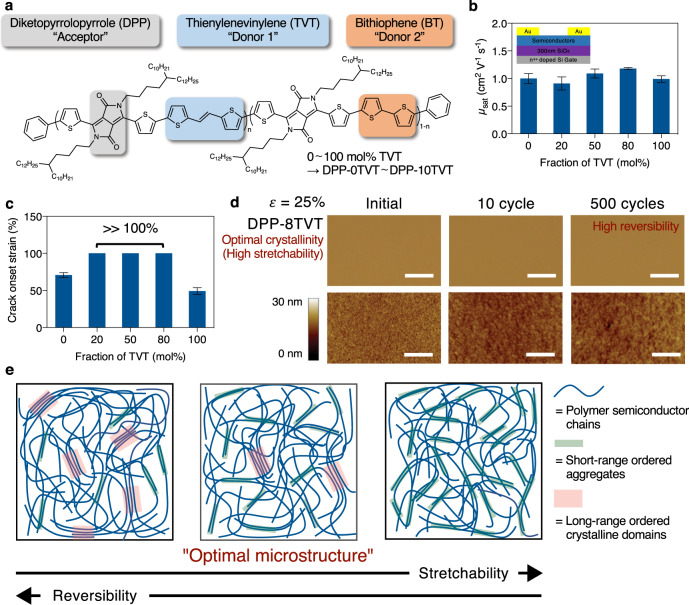
Molecular design and properties of stretchable terpolymers. **a** Chemical structure of terpolymers used in this study. Based on the TVT unit fraction, terpolymers were named as DPP-0TVT (0 mol% TVT) ~ DPP-10TVT (100 mol% TVT). **b** Mobility of the terpolymers measured from top-contact-bottom-gate transistors given in the top left corner (measured in air). Error bars represent SD. **c** Crack onset strain of the terpolymers. Our terpolymers showed tremendous crack onset strain >100% strain. Error bars represent SD. **d** Optical and AFM images of 30 nm-thick semiconducting terpolymer films after repeated 25% strain. DPP-8TVT showed high mechanical reversibility without wrinkling. The color scale of the AFM images represents relative height (scale bar: 10 µm and 1 µm for optical and AFM images, respectively). **e** Microstructures of polymer semiconductors that are ideal for stretchability and mechanical reversibility. Semi-crystalline polymer semiconductors with low crystallinity (such as DPP-8TVT) have optimal microstructures with balanced stretchability and mechanical reversibility.

In this work, we prepared a series of diketopyrrolopyrrole-based (3,6-bis(5-bromothiophen-2-yl)-2,5-bis(4-decylhexadecyl)-2,5-dihydropyrrolo[3,4-c]pyrrole-1,4-dione (DPP)-based) terpolymer semiconductors with different fractions of thienylenevinylenethiophene ((*E*)-1,2-bis(5-(trimethylstannyl)thiophene-2-yl)ethene ((TVT)) and bithiophene (BT)-conjugated co-monomer units (i.e., the donor units) (Fig. 1a, Supplementary Fig. 1, and Supplementary Table 1). Such terpolymers were labeled as DPP-0TVT (0 mol% TVT and 100 mol% of BT) to DPP-10TVT (100 mol% TVT and 0 mol% of BT), with their molecular weight and dispersity summarized in Supplementary Table 2.

In our molecular design, we hypothesized that the use of structurally similar co-monomers may be essential to maintain short-range aggregation. If the co-monomers are significantly different, then the short-range aggregation may be disrupted, which may result in reduced mobility. For example, Lin et al.^40^ reported significant decrease in mobility was observed in terpolymer semiconductors, when the polymers were prepared with dissimilar co-monomers. As TVT and BT have similar structures, such mobility compromise may be avoided in our terpolymers.

First, the electrical and mechanical properties of the terpolymers were measured. As hypothesized, all the terpolymers showed mobility values >1 cm^2^ V^−1^ s^−1^, comparable to or even greater than those of the regular copolymers (DPP-0TVT and 10TVT) (Fig. 1b and Supplementary Figs. 3–5). Importantly, all terpolymers exhibited improved stretchability with crack onset strain >100% regardless of TVT fraction (Fig. 1c and Supplementary Figs. 6–9). These results show that our simple molecular design effectively mitigated the long-standing trade-off between mobility and stretchability. Also, the high mobility and stretchability of DPP-8TVT was shown to be independent of annealing temperature and time (Supplementary Fig. 10). Interestingly, the modulus of the terpolymers showed little correlation with stretchability (Supplementary Figs. 11 and 12), which we attributed to the fully conjugated backbone with similar chemical structures. In addition to high stretchability and mobility of the terpolymers, their air stability was also examined. As shown in Supplementary Fig. 13, no significant change of transistor characteristics was observed, which indicates that our terpolymers are air stable up to 30 days. DPP-8TVT was used for additional characterizations and to understand the underlying mechanisms for our observed improvement in mechanical and electrical properties, as it simultaneously exhibited the highest mobility and stretchability.

**Fig. 2:**
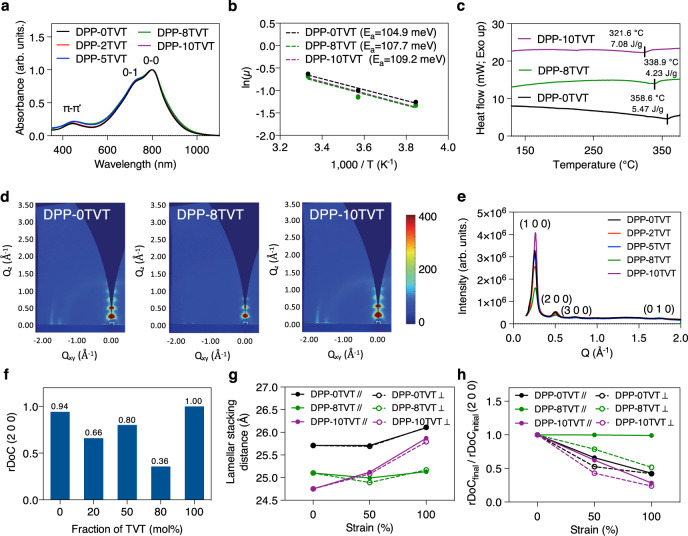
Characterization of stretchable terpolymers. **a** UV-Vis/NIR absorption spectra of the 30 nm-thick terpolymer films prepared by spin coating from chlorobenzene followed by drying at 150 °C for 15 min. **b** Activation energy for charge transport of DPP-0TVT, 8TVT, and 10TVT. The activation energy was extracted from the slope. *μ* is the charge carrier mobility extracted from saturation mobility of transistor measurements. **c** DSC second scan of DPP-0TVT, 8TVT, and 10TVT. **d** GIXD images of thin films (30 nm) of DPP-0TVT, 8TVT, and 10TVT. DPP-8TVT clearly showed higher-order diffraction peaks with smaller intensity. The color scale of the GIXD images represents relative diffraction intensity. **e** Diffraction intensity along *Q*_z_ direction vs. *Q*-values for terpolymers from GIXD measurements. **f** Relative degree of crystallinity (rDoC) of the terpolymers, which was calculated based on (2 0 0) peaks. **g** Lamellar stacking distance change under strain. **h** rDoC change under strain.

Second, an important parameter of stretchable semiconductors is mechanical reversibility against cyclic strain. As stretchable electronic devices will experience unexpected and repeated mechanical deformations, maintaining their functionalities against cyclic strain is necessary. However, the fundamental mechanisms of mechanical reversibility may be different from those of other polymer systems. We hypothesized that the crystalline domains of our semiconducting polymers may act as physical crosslinking sites, which may provide the terpolymers high mechanical reversibility. As crystalline domains may be fractured under strain and reduce stretchability^41^, it is thus important to achieve “optimal” microstructures to enable both high stretchability and mechanical reversibility. From optical and atomic force microscope (AFM) images shown in Fig. 1d, DPP-8TVT remained free from mechanical damages and wrinkles even after cycling 500 times between 0 and 25% strain. We compared DPP-8TVT with semiconducting polymers with crystalline domains of high orders (DPP-0TVT and DPP-10TVT, Fig. 2d) and a literature reported “near-amorphous” semiconducting polymer based on indacenodithiophene–benzothiadiazole (IDTBT)^41^ (Supplementary Fig. 14). It was observed that the more crystalline semiconductors (DPP-0TVT and DPP-10TVT) experienced severe fracturing at 25% strain, whereas the near-amorphous IDTBT only showed significant wrinkling, but no cracks could be observed (Supplementary Figs. 15–19). These results indicated that DPP-8TVT contained sufficient crystallinity for high mechanical reversibility, whereas the crystallinity is low enough for high stretchability (Fig. 1e).

### Controlled multi-scale ordering of the terpolymers

To understand the mechanisms of our terpolymer design achieving both high mobility and great mechanical properties, we first performed AFM to characterize the surface morphology of the polymer thin films. We observed that our terpolymers have fiber-like morphology, which is commonly observed for such conjugated polymers^42^ (Supplementary Fig. 20). Next, ultraviolet-visible/near-infrared (UV-Vis/NIR) spectroscopy was performed to understand the intermolecular aggregation of the terpolymers. As conjugated polymer semiconductors often form aggregates, comparing the intensity of 0-1 and 0-0 vibronic peaks can offer information regarding the relative degree of aggregation^43,44^. In both solution and thin-film states, negligible change of relative degree of aggregation was observed from DPP-0TVT to DPP-10TVT (Fig. 2a and Supplementary Fig. 21), which confirmed our hypothesis that the terpolymers are able to maintain local short-range aggregates. As aggregation is essential for efficient inter-chain charge transport, the obtained results explained the relatively unchanged mobilities of our terpolymers. The activation energy for charge transport was extracted from temperature-dependent electrical measurements using the Arrhenius equation^45^. DPP-8TVT showed similar charge transport activation energy, suggesting no change in trap states compared to other polymers (Fig. 2b and Supplementary Fig. 22).

Next, the thermal stability of the polymers was characterized by thermal gravimetric analysis (Supplementary Fig. 23). All polymers were found to be thermally stable without decomposition at temperatures <400 °C. We then performed differential scanning calorimetry (DSC) below the determined decomposition temperature to observe any thermal transitions of our polymers. The heat of fusion of each semiconductor was extracted from the melting transition in the second heating scan observed near 340–360 °C (Fig. 2c), from which relative crystallinity information can be extracted. DPP-8TVT clearly showed a lower heat of fusion, which indicates that its crystallinity is the lowest followed by DPP-0TVT and 10TVT.

To understand how microstructures of the polymer thin films are affected by the terpolymer design, we performed grazing incidence X-ray diffraction (GIXD). Interestingly, the diffraction intensity of the terpolymers was significantly lower than that of the reference copolymers (DPP-0TVT and 10TVT) (Fig. 2d and Supplementary Fig. 24). The relative degree of crystallinity (rDoC) of the films was calculated based on (2 0 0) diffraction peak intensity. Consistent with the DSC data, the terpolymers showed clearly lower rDoC than the regular copolymers DPP-0TVT or DPP-10TVT (Fig. 2e, f). This result confirms our hypothesis that the structural randomness of the terpolymers may decrease the crystallinity, i.e., the longer-range order^39^. Interestingly, DPP-8TVT showed the lowest crystallinity, although it contains the rigid unit (TVT) more than other terpolymers (DPP-2TVT and 5TVT). The backbone conformation of the terpolymers may be highly disrupted, as the two different co-monomers are randomly incorporated. In this case, the more flexible unit (BT) may be able to easily fill in the structural defects to form ordered domains, whereas the more rigid unit (TVT) may not.

The detailed parameters of the semiconductor films from GIXD experiments are summarized in Supplementary Table 3. Interestingly, the terpolymers exhibited larger full width at half maximum values than the reference copolymers in both lamellar and *π*–*π* stacking peaks, which indicates that the coherence length of the crystalline ordered domains is shorter in the terpolymers. Together with the obtained information about aggregation from UV-Vis/NIR, we conclude that our terpolymers contain the comparable degree of short-range order with decreased longer-range crystalline domains. The above terpolymer morphology is the key to achieving high stretchability without compromising mobility.

Another important parameter is the glass transition temperature (*T*_g_) of the terpolymers, as dynamic behaviors of the polymeric chains may affect the microstructures and the properties. In our terpolymer design, we expected that the difference in *T*_g_ may be negligible, given the structural similarity of the co-monomers used to construct the terpolymers (Supplementary Fig. 25). Therefore, the improved stretchability might not originate from the enhanced dynamic behaviors of the polymeric chains, but from the controlled multi-scale ordering.

In stretchable electronics applications, our terpolymer semiconductors may be used under strain, which requires understanding the microstructure change in the stretched terpolymer semiconductor films. Several mechanisms can result in dissipating applied mechanical stress, such as chain stretching and alignment in amorphous domains, breaking aggregates and/or crystalline domains, rotation of monomer units, and breaking weaker bonding (i.e., hydrogen bonding or metal–ligand bonding)^32,34,46–48^. We hypothesized that our terpolymers with low degree of crystallinity and lack of large crystalline domains may effectively dissipate applied mechanical stress without affecting their crystalline domains. On the other hand, it is more likely that crystalline domains will break under strain for semiconducting polymers with high crystallinity or large crystalline domains, which is not fully reversible. We characterized the change of crystalline domains by examining the following three parameters from GIXD data: lamellar stacking distance and rDoC extracted from (2 0 0) and (0 1 0) peaks. DPP-8TVT exhibited the least change of the above three parameters under strain (Fig. 2g, h, Supplementary Figs. 26–29, and Supplementary Table 4), which suggests that the DPP-8TVT crystalline domains showed the smallest deformation, i.e., a little change of lamellar stacking distance and the fracture of crystalline domains was not much necessary (small change of rDoC). In other words, DPP-8TVT maintained its crystalline domains relatively well under strain. Interestingly, significant anisotropy in rDoC was observed under strain (Fig. 2h and Supplementary Fig. 29), which we attributed to strain-induced chain alignment. As shown in Supplementary Fig. 8, DPP-8TVT chains showed the most significant degree of alignment under strain. Thus, the decrease in rDoC of DPP-8TVT in the direction perpendicular to the strain might arise from the chain alignment effect.

### Comparison with polymer blends

**Fig. 3:**
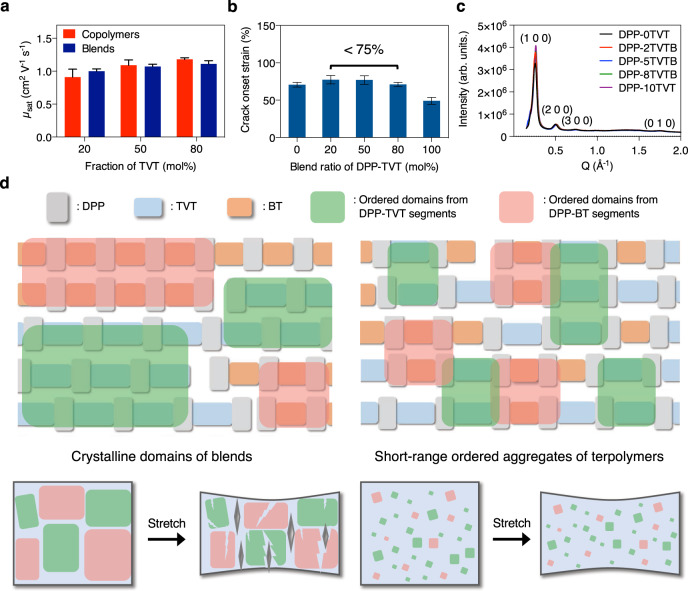
Comparison of polymer blend and terpolymer films. **a** Mobilities of the polymer blend films and the terpolymer ones measured from top-contact-bottom-gate transistors given in Fig. 1c. Error bars represent SD. **b** Crack onset strain of the polymer blends. The blend films showed negligible improvement of stretchability. Error bars represent SD. **c** Diffraction intensity along *Q*_z_ direction vs. *Q*-values of the polymer blend films from GIXD measurements. The blend films showed negligible intensity change. **d** Schematic of the proposed mechanisms behind different behaviors of the blend and the terpolymer films under strain.

One question that may arise is whether the simple blending of DPP-0TVT and DPP-10TVT can achieve the same thin-film microstructures, and therefore similar electrical and mechanical properties as DPP-8TVT. The two different monomer units (TVT and BT) in our terpolymers are randomly and covalently bonded to each other. On the other hand, the two different copolymer chains in a blend may be unable to mix at the molecular level. We even observed enhanced aggregation formation due to blending previously^49^. Therefore, we hypothesized that the blend films may not show improvement in stretchability. We observed that in terms of charge transport, the terpolymer and blend films showed comparable mobilities (Fig. 3a and Supplementary Fig. 30). However, unlike the terpolymer films, the blend ones exhibited negligible enhancement of stretchability (Fig. 3b and Supplementary Fig. 31). To confirm our hypothesis that the unchanged crystallinity of the blend films is responsible for its low stretchability, we next performed GIXD measurements. The blends maintained similar crystallinity as the neat polymers (Fig. 3c, Supplementary Fig. 32, and Supplementary Table 5). On the other hand, the overall degree of short-range aggregates in the blend films was observed to be comparable to our reference copolymers and terpolymers from UV-Vis/NIR (Supplementary Fig. 33). These results support our hypothesis that controlling the microstructures is the key to simultaneously achieving high mobility and stretchability. Although both crystalline domains and short-range ordered aggregates can facilitate efficient charge transport, large crystalline domains may be more susceptible to mechanical damages induced by strain than the short-range aggregates (Fig. 3d).

### General applicability

**Fig. 4:**
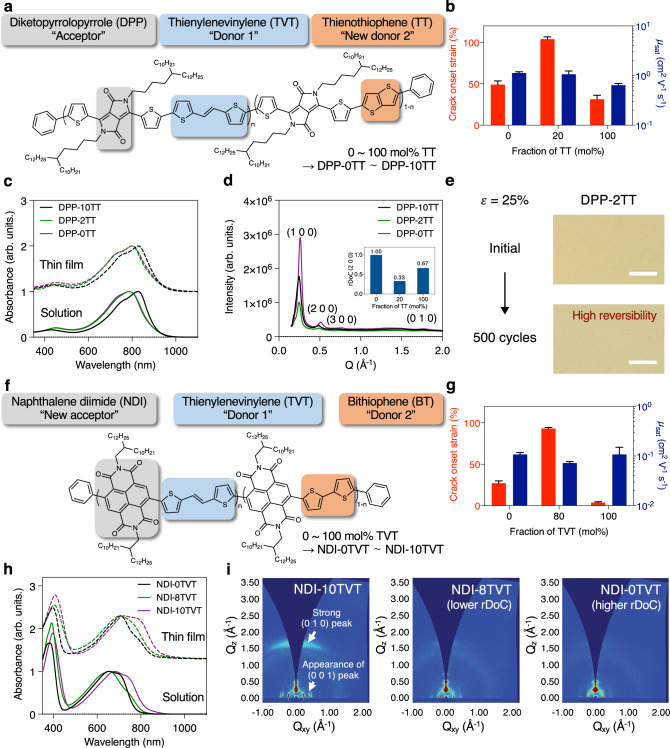
General applicability of our terpolymer-based stretchable semiconductor design. **a** Chemical structure of conjugated polymers based on TVT and TT units as donors. **b** Crack onset strain and mobility of the polymer semiconductors (measured in air). Error bars represent SD. **c** UV-Vis/NIR absorption spectra of the semiconducting polymers in thin-film and solution states. In both cases, our terpolymer showed well-maintained overall ordering. **d** Intensity vs. *Q* graph extracted from GIXD measurements. **e** Optical images of DPP-2TT films before and after 500 times of 25% strain. DPP-2TT showed excellent mechanical reversibility without wrinkling (scale bar: 10 µm). **f** Chemical structure of *n*-type conjugated polymers based on the NDI unit as an acceptor. **g** Crack onset strain and mobility of the *n*-type polymer semiconductors (measured in nitrogen). Error bars represent SD. **h** UV-Vis/NIR absorption spectra of the *n*-type semiconducting polymers in thin-film and solution states. **i** GIXD images of the *n*-type polymer semiconductors.

Another important advantage of our molecular design is that various combinations of electron donors and acceptors can be used to construct terpolymer semiconductors or even more than three types of units in the copolymers. To demonstrate the general applicability of our terpolymer design, two additional terpolymer semiconductors (one *p*- and one *n*-type) with different donor and acceptor combinations were prepared. We used 80 mol% of TVT for all of the terpolymers, which showed the best stretchability and mobility in our initial test system. Once TVT and thienothiophene (5,5′-bis(trimethylstannyl)-2,2′-BT, 2,5-bis(trimethylstannyl)thieno[3,2-b]thiophene (TT)) were used as monomers (Fig. 4a), crack onset strain was significantly improved from 30% to 105% with no degradation of mobility (Fig. 4b and Supplementary Figs. 34 and 35). Notably, the mechanism to control multi-scale ordering was still applied to this system. With UV-Vis/NIR and GIXD measurements, we observed that this terpolymer with 80 mol% TVT and 20% TT again exhibited well-maintained short-range aggregates (Fig. 4c), whereas its crystallinity was significantly reduced (Fig. 4d, Supplementary Fig. 36, and Supplementary Table 6). The optimal microstructures of the terpolymer again resulted in high mechanical reversibility (Fig. 4e).

Moreover, an intrinsically stretchable *n*-type semiconducting polymer was developed utilizing our molecular design principle (Fig. 4f and Supplementary Fig. 2). So far, few such polymers have been reported^50^. When naphthalene diimide (4,9-dibromo-2,7-bis(2-decyltetradecyl)benzo[lmn][3,8]phenanthroline-1,3,6,8(2H,7H)-tetraone ((NDI)) was used as an acceptor unit, our newly developed NDI-8TVT exhibited the crack onset strain of 93%, which was a 30-fold improvement compared with NDI-10TVT. Even with such a high crack onset strain, the mobility of the NDI-8TVT was comparable to the same values of NDI-0TVT and 10TVT (Fig. 4g and Supplementary Figs. 37–40). In addition, NDI-8TVT exhibited high mechanical reversibility (Supplementary Fig. 41). With UV-Vis/NIR and GIXD measurements, we found that NDI-8TVT showed the lowest rDoC with well-maintained short-range ordered aggregates, comparable to that of the two reference polymers (NDI-0TVT and 10TVT) (Fig. 4h, i). These results again confirm that the terpolymer semiconductors may achieve high stretchability with significantly reduced crystallinity, whereas a high fraction of short-range ordered aggregates may result in high mobility. All of the above examples suggest our molecular design can be used to develop various intrinsically stretchable polymer semiconductors (both *p*- and *n*-type) with the same underlying mechanism of well-controlled microstructures. We expect that the mobility and stretchability of terpolymer semiconductor films may be further improved with new sets of electron donors and acceptors.

All of the above results indicate that our terpolymers possess all of the three desired properties (high mobility, stretchability, and mechanical reversibility) as stretchable polymer semiconductors. Importantly, the terpolymer design has significantly improved reliability, compared with previously reported multi-component stretchable semiconductor films with elastomers or molecular additives. Specifically, significant sample-to-sample variations occur when the second component is introduced, depending on the choice of the second component, its fraction, and processing conditions, which limits the applications of such approaches^36,37^. However, our terpolymers exhibited significantly improved stretchability without any compromise of mobility, regardless of processing conditions (Supplementary Fig. 10) and molecular components (Figs. 1 and 4). In addition, our terpolymer-based stretchable semiconductors are easily achievable with simples synthesis, which distinguishes this work from literature reported approaches that require additional synthetic steps^32,33^.

### Fully stretchable transistors

**Fig. 5:**
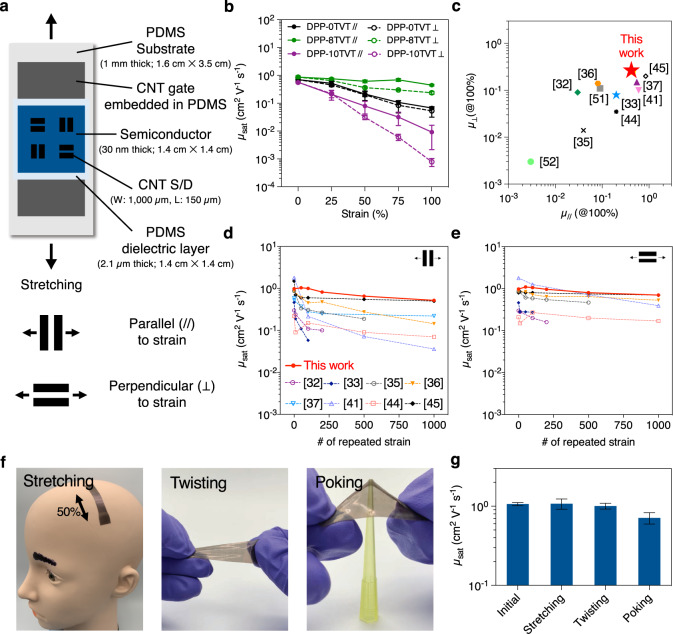
Fully stretchable transistors and their applications. **a** Schematic structure of fully stretchable transistors fabricated with our polymer semiconductors. Carbon nanotubes (CNTs) were used for source, drain, and gate electrodes. Polydimethylsiloxane (PDMS) was used for substrates and dielectrics. **b** Mobility change of DPP-0TVT, 8TVT, and 10TVT under strain. DPP-8TVT showed significantly greater mobility retention under strain. Error bars represent SD. **c** Mobility of DPP-8TVT under 100% strain compared with those of stretchable semiconducting polymers measured in stretchable transistors reported in the literature. Mobility of DPP-8TVT after repeated strain compared with the same value of stretchable semiconducting polymers measured in stretchable transistors reported in the literature in the direction **d** parallel and **e** perpendicular to strain. **f** Photos of the fully stretchable transistors under various mechanical deformations. **g** Mobility of the fully stretchable transistors after the mechanical deformations. Error bars represent SD.

Last, we fabricated fully stretchable transistors with our newly established terpolymer semiconductor films (DPP-8TVT) (Fig. 5a). As DPP-8TVT maintained its ordered domains under strain (Fig. 2g, h), the terpolymer was expected to maintain its mobility well under strain. Also, due to its high mechanical reversibility, we expect the mobility will not degrade significantly against repeated mechanical deformation. Indeed, DPP-8TVT showed significantly greater mobility retention under strain than DPP-0TVT and 10TVT (Fig. 5b and Supplementary Figs. 42 and 43). When DPP-8TVT is compared with other stretchable polymer semiconductors reported so far, it shows one of the highest mobility values under 100% strain^45,51,52^ (Fig. 5c and Supplementary Table 7). The mobility of DPP-8TVT was also measured after repeated 25% strain. DPP-8TVT showed a negligible decrease in mobility even after 1000 times of 25% strain, which is one of the highest mobility retention capabilities against repeated strain compared with other reported stretchable polymer semiconductors (Fig. 5d, e and Supplementary Fig. 44). All of these obtained results confirm that our terpolymer is among the best-performing stretchable semiconducting polymers reported so far. Our fully stretchable transistors were then exposed to various mechanical deformations, such as stretching, twisting, and poking with a sharp object, which they may experience in daily usages (Fig. 5f). Even after those mechanical deformations, our fully stretchable transistors showed little mobility degradation, which indicates the high robustness of terpolymer films (Fig. 5g and Supplementary Fig. 45). In order to show the high scalability of our terpolymers, fully stretchable transistor arrays were then fabricated. The stretchable transistors fabricated with DPP-8TVT exhibited high uniformity in mobility (Supplementary Fig. 46).

## Discussion

In conclusion, we have successfully developed terpolymer-based stretchable polymer semiconductors. Our described molecular design is readily achievable with various monomers and therefore the stretchable polymer semiconductors are easily synthesized. The newly designed terpolymer semiconductors show high stretchability and mechanical reversibility, while retaining high charge carrier mobility even after repeated strain. Through the detailed characterization experiments, it was shown that the terpolymers maintained short-range aggregations with reduced overall crystallinity and average crystalline domain sizes. Furthermore, a series of intrinsically stretchable semiconductors, including several *p*- and *n*-type structures, were demonstrated using our molecular design concept. The fully stretchable transistors fabricated with our terpolymer films do not suffer from performance degradation even after being subjected to repeated or various types of mechanical deformations. To the best of our knowledge, the fabricated stretchable transistors exhibited one of the highest mobilities under repeated strain. With fine-tuning of additional molecular parameters, such as monomer choice, fraction, and backbone sequence, we believe terpolymer-based semiconductors can be further improved, providing a promising path towards high-performance stretchable polymer semiconductors.

## Methods

### Materials

All reagents were purchased from Sigma-Aldrich and TCI America. Tris(dibenzylideneacetone)-dipalladium(0)-chloroform adduct (Pd_2_(dba)_3_CHCl_3_) was recrystallized from acetone, then Pd_2_(dba)_3_CHCl_3_ crystal was collected by filteration^53^. DPP monomer was successfully synthesized from 3,6-di(2-thienyl)-2,5-dihydropyrrolo[3,4-*c*]pyrrole-1,4-dione with branched alkyl iodide compound via *N*-alkylation and is followed by the bromination with *N*-bromosccineimide^43^. NDI monomer was synthesized from 2,6-dibromonaphthalene-1,4,5,8-tetracarboxylic dianhydlide with 2-decyltetradecane-1-amine^54^. TVT, TT, and the other commercial reactants were used without further purification. Polydimethylsiloxane (PDMS) precursors (Sylgard 184) were purchased from Dow Corning. Isopropanol and ammonium hydroxide were purchased from Fischer Scientific. Poly(3-hexylthiophene) (P3HT) was provided by BASF. Carbon nanotubes (CNTs) were purchased from Carbon Solutions, Inc. All of the above materials were used without further purification for substrate and device fabrication.

### Synthesis of various conjugated polymers

All of the studied conjugated polymers based on DPP and NDI main-chain structures were synthesized by the Stille coupling polycondensation using microwave reactor with dibrominated DPP or NDI monomer, and stannylated compounds (TVT, BT, and TT)^43^. For the polymer synthesis, all of the reactions were carried out by using the Biotage® Initiator+ Microwave System. Details of all synthetic procedures are available in the Supplementary Information (Supplementary Figs. 1 and 2, and Supplementary Table 1). Molecular weight and dispersity of the polymers are given in Supplementary Table 2.

### Materials characterization

Wafers with 300 nm silicon dioxide on n^++^ Si were purchased from Namkang Hi-Tech. The SiO_2_ surface was functionalized with octadecyltrimethoxysilane (OTS) according to our reported method^33^. Semiconductor polymer solutions (4–5 mg mL^−1^ in chlorobenzene) were spin-coated on OTS-treated SiO_2_ wafers at 1200 r.p.m. for 1 min. The obtained films (30 nm in thickness) were annealed at 150 °C for 15 min.

Gold was deposited on the obtained films as the source and drain electrodes (channel width = 1000 µm and channel length = 50 µm). Mobility of the conjugated polymer films was determined using Keithley 4200 in air (DPP-based semiconductors) or in nitrogen environments (NDI-based semiconductors). Then, 10 nF cm^−2^ was used as the capacitance of the 300 nm SiO_2_ dielectric layer. For activation energy characterization, the devices were measured at three different temperatures (360, 280, and 300 K) under vacuum.

The mechanical properties of the conjugated polymers were determined using a film-on-elastomer method. Polymer films were first transferred onto PDMS substrates (precursor:crosslinker = 12:1). The films were strained to observe crack formation. The elastic modulus of the films was characterized through a buckling method. The polymer films were transferred onto pre-strained PDMS substrates. Once the strain was released, wrinkles were clearly formed, whereas elastic modulus of the films was derived from the wrinkle wavelength and film thickness. Finally, the reversibility of the polymer films was characterized by observing wrinkle formation after repeated strain.

Absorption of the thin films was studied using UV-Vis/NIR spectroscopy in the range of 350–1100 nm (Agilent Cary 6000i UV-Vis/NIR spectrometer). For dichroic ratio measurements, a polarizer was used in the direction parallel and perpendicular to the applied strain. The dichroic ratio is defined as A_//_/A_⊥_, where A_//_ and A_⊥_ are absorbance values in the strain direction and the same value in the direction perpendicular to the strain.

GIXD was performed at beamline 11-3 of Stanford Synchrotron Radiation Lightsource (SSRL). The thin films were exposed to X-ray for 5 min with an incidence angle of 0.12°. The rDoC was derived from (2 0 0) peaks of the thin films.

The thermal properties of the polymers were characterized by DSC. For this measurement, bulk polymer samples were loaded into DSC pans. From heat flow, the melting temperature and the heat of fusion of our polymer samples were characterized.

### Stretchable transistors

CNT (P2-SWNT, Carbon Solutions, Inc., 0.2 mg mL^−1^) and P3HT (from BASF, 0.05 mg mL^−1^) in chloroform were ultrasonicated for 15 min at 30% amplitude using a 750 W probe. The dispersed CNT was then centrifugated at 503.1 × *g* for 15 min. The dispersed CNT/P3HT mixture was spray-coated on OTS-treated SiO_2_ wafers using a commercial airbrush (Master Airbrush, SB844). PDMS (precursor:crosslinker = 12:1) was directly spin-coated on the CNT/P3HT mixture. This CNT/P3HT/PDMS composite was annealed at 80 °C overnight and used as a gate electrode. PDMS dielectric solution (250 mg mL^−1^, precursor:crosslinker = 12:1, in hexane) was filtered using a 1 µm glass microfiber filter. The PDMS solution was spin-coated directly onto conjugated polymer films at 5000 r.p.m. for 2 min. The PDMS dielectric films (2.1 µm in thickness) were then annealed at 150 °C for 2 h. The dielectric/semiconductor layers were transferred on the gate electrode. For source/drain electrodes, CNT (P3-SWNT, Carbon Solutions, Inc., 0.3 mg mL^−1^ in isopropanol) was sonicated overnight. The CNT mixture was then ultrasonicated and centrifugated at the same conditions used for P2-SWNT. The dispersed CNT was directly spray-coated onto the semiconducting layer of a fully stretchable transistor. Shadow masks with a channel length of 150 µm and a channel width of 1000 µm were used for spray-coating. All of the above fabrication processes were performed under ambient conditions.

### Fabrication of intrinsically stretchable transistor arrays

A SiO_2_/Si wafer was cleaned with oxygen plasma (150 W, 200 mTorr) for 2 min and then sonicated in acetone, 2-propanol, and deionized water for 5 min each. Dextran (10 wt% in water) was spin-coated on top of cleaned Si/SiO_2_ wafer at 1200 r.p.m. for 15 s. Next, it was baked at 180 °C for 30 min in ambient conditions to fully remove the water. SEBS (H1052 from Asahi Kasei) solution (80 mg/ml in Toluene) with azide crosslinker (2.0 mg/ml) was spin-coated on top of dextran at 1000 r.p.m., to form the dielectric layer with 2 µm thickness. The film was then selectively exposed to deep-UV light (254 nm wavelength, Spectrum 1000 Precision UV Spot Curing System from American Ultraviolet) with a mask for 10 min with a dose of ~1080 mJ cm^−2^. After this, it was baked at 120 °C for 15 min in air, and subsequently developed in dodecane for 45 s to remove the unexposed parts. To fully crosslink the film, it was further baked at 200 °C for 1 h in glovebox. Then, DPP-8TVT films were transferred onto the patterned dielectric layer by using a PDMS stamp. To pattern the semiconductor, a fluorinated layer (3 M™ Novec™ 1902 Electronic Grade Coating diluted by ethoxynonafluorobutane in 1:2 volume ratio) was spin-coated on the DPP-8TVT films at 1000 r.p.m. for 1 min. Then, a Cu etching mask (150 nm) was thermally evaporated on top with a shadow mask. The semiconductor without Cu on top was then etched away by O_2_ plasma (150 W, 200 mTorr), followed by lifting-off the Cu film in ethoxynonafluorobutane. Then, source/drain electrodes were patterned by spray-coating CNTs (P3-SWNT in isopropanol) with a shadow mask. Next, SEBS (H1221 from Asahi Kasei) stretchable substrate (180 µm in thickness) was laminated on top and the whole device was soaked into water to dissolve the dextran sacrificial layer and transfer all components onto the substrate. Finally, CNTs (P3-SWNT in isopropanol) were spray-coated through a mask to get gate electrodes on the SEBS dielectric layer. After entire processes, the fabricated transistor arrays were baked at 70 °C under vacuum for 4 h to remove any moisture.

## Supplementary information

Supplementary Information

## Data Availability

The authors declare that the main data supporting the findings of this study are available within the article and its Supplementary Information. Extra data are available from the corresponding author (Z.B.) upon request.
