# Supplementary material for: A design strategy for high mobility stretchable polymer semiconductors
Source: Nat Commun. 2021 Jun 11;12:3572. doi: 10.1038/s41467-021-23798-2 (PMC8196107; doi:10.1038/s41467-021-23798-2)
Supplement: Supplementary file 1 — Supplementary Information [file 41467_2021_23798_MOESM1_ESM.pdf]

Supplementary Information

A design strategy for high mobility stretchable polymer semiconductors

Jaewan Mun[1,7], Yuto Ochiai[1,2,7], Weichen Wang[3], Yu Zheng[4], Yuqing Zheng[1], Hung-Chin Wu[1,], Naoji Matsuhisa[1,6], Tomoya Higashihara[2], Jeffrey B.-H Tok[1], Youngjun Yun[5*], Zhenan Bao[1*]

[1]Department of Chemical Engineering, Stanford University, Stanford, CA 94305, USA

[2]Department of Organic Materials Science, Yamagata University, 4-3-16 Jonan, Yonezawa, Yamagata 992-8510, Japan.

[3]Department of Materials Science and Engineering, Stanford University, Stanford, CA 94305, USA

[4]Department of Chemistry, Stanford University, Stanford, CA 94305, USA

[5]Samsung Advanced Institute of Technology, 130 Samseong-ro, Suwon, 16678, South Korea

[6]Present address: Department of Electronics and Electrical Engineering, Keio University, 3-14-1 Hiyoshi, Kohoku-ku, Yokohama 223-8522, Japan

[7]These authors contributed equally: J. Mun, Y. Ochiai.

[*]Corresponding Authors, Email: youngjun.yun@gmail.com & zbao@stanford.edu

Contents

1. General Synthetic procedure of semiconducting polymers
2. Mechanical and electrical properties of prepared polymers
3. Mechanisms behind the improved stretchability without compromise in mobility
4. Microstructure evolution of conjugated polymer films under strain
5. Comparison of our terpolymers with blends
6. General applicability of our terpolymer design for developing stretchable polymer semiconductors
7. Fully stretchable transistors and demonstrations
8. Reference

1. General Synthetic procedure of semiconducting polymers

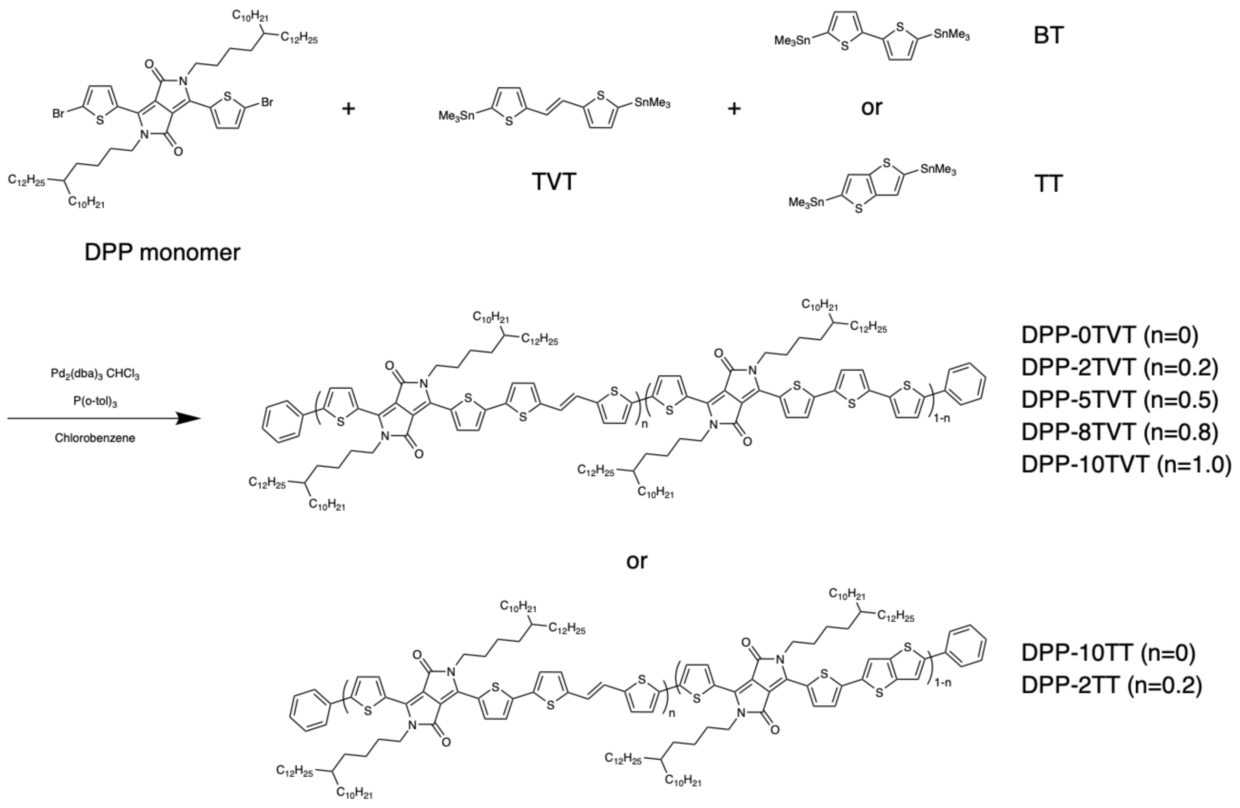

Supplementary Figure 1. Synthetic pathway of various DPP-based polymers. DPP-based semiconducting polymer were synthesized by the Stille coupling polycondensation.

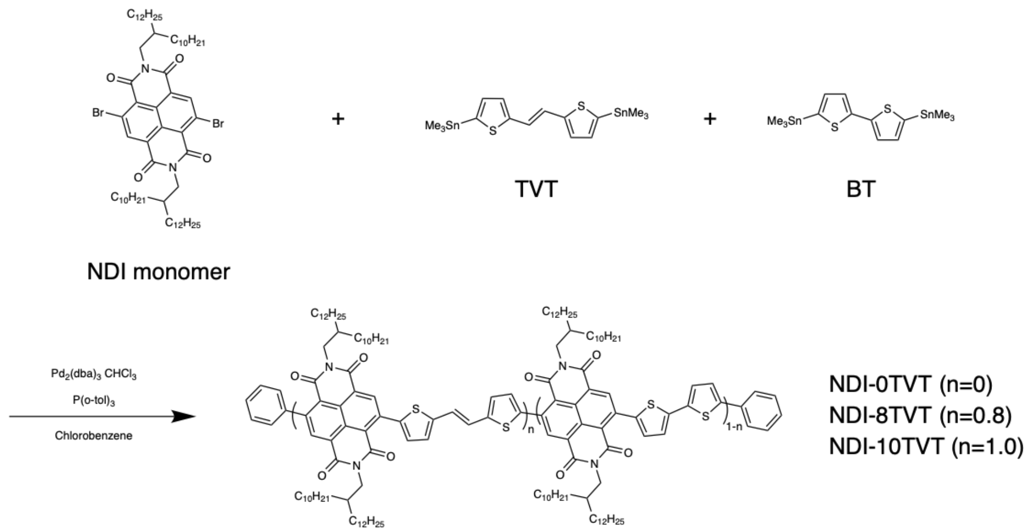

Supplementary Figure 2. Synthetic pathway of various NDI-based polymers. NDI-based semiconducting polymer were synthesized by the Stille coupling polycondensation.

Supplementary Table 1. Molar equivalence of monomers. Molar equivalence of the monomers used for various polymerizations are summarized.

Polymer	Acceptor unit (A)	Donor unit (D1)	Donor unit (D2)	Molar equivalence (equiv.)		
				A	D1	D2
DPP-0TVT	DPP	-	BT	1.0	-	1.0
DPP-2TVT	DPP	TVT	BT	1.0	0.2	0.8
DPP-5TVT	DPP	TVT	BT	1.0	0.5	0.5
DPP-8TVT	DPP	TVT	BT	1.0	0.8	0.2
DPP-10TVT (DPP-0TT)	DPP	TVT	-	1.0	1.0	-
DPP-10TT	DPP	-	TT	1.0	-	1.0
DPP-2TT	DPP	TVT	TT	1.0	0.8	0.2
NDI-0TVT	NDI	-	BT	1.0	-	1.0
NDI-8TVT	NDI	TVT	BT	1.0	0.8	0.2
NDI-10TVT	NDI	TVT	-	1.0	1.0	-

Synthesis of the various DPP-based polymers:

To a reaction vessel, 3,6-bis(5-bromothiophen-2-yl)-2,5-bis(4-decylhexadecyl)-2,5-dihydropyrrolo[3,4-c]pyrrole-1,4-dione (DPP monomer) (0.1 g, 0.082 mmol, 1 equiv.), (*E*)-1,2-bis(5-(trimethylstannyl)thiophene-2-yl)ethene (TVT), 5,5'-bis(trimethylstannyl)-2,2'-bithiophene (BT) or 2,5-bis(trimethylstannyl)thieno[3,2-b]thiophene (TT), recrystallized Tris(dibenzylideneacetone)-dipalladium(0)-chloroform adduct ($Pd_2(dba)_3CHCl_3$) (1.7 mg, 2.22 mmol, 0.02 equiv.),$CHCl_3$, and P(*o*-tol)$_3$ (2.0 mg, 0.007 mmol, 0.08 equiv.) were dissolved in 3 mL of chlorobenzene and sealed in the nitrogen-filled glovebox. The reaction vessel was subjected to Biotage® Initiator+ Microwave System with the following temperature profile, 2 mins at 100 °C, 2 mins at 120 °C, 5 mins at 140 °C, 5 mins at 160 °C and 40 mins at 180 °C. After the polymerization, excess amount of the trimethyl(phenyl)stannane was injected into the crude polymer solution and heated 150 °C for 10 min. After completion, excess amount of bromobenzene was injected into the reaction vessel and heated 150 °C for 10 min. The crude polymer was precipitated in methanol, collected by filtration, then loaded to an extraction thimble. The thimble was left into a soxhlet extractor, and the crude polymer was successively washed with methanol, acetone, and hexane. The resulting polymer was collected by chlorobenzene. The chlorobenzene solution with extracted polymer was concentrated and then precipitated into methanol. The precipitated polymer was collected by filtration and dried under high vacuum overnight, yielding the various studied DPP-based copolymers as dark green solid.

Synthesis of the various NDI-based polymers:

To a reaction vessel, 4,9-dibromo-2,7-bis(2-decyltetradecyl)benzo[lmn][3,8]phenanthroline-1,3,6,8(2H,7H)-tetraone (NDI monomer) (0.1 g, 0.091 mmol, 1 equiv.), TVT, BT, recrystallized $Pd_2(dba)_3CHCl_3$ (2.0 mg, 0.019 mmol, 0.02 equiv.),, and P(*o*-tol)$_3$ (2.3 mg, 0.076 mmol, 0.08 equiv.) were dissolved in 3 mL of chlorobenzene and sealed in the nitrogen-filled glovebox. The reaction vessel was subjected to microwave system with the following temperature profile, 2 mins at 100 °C, 2 mins at 120 °C, 5 mins at 140 °C, 5 mins at 160 °C and 40 mins at 180 °C. After the polymerization, the excess amount of trimethyl(phenyl)stannane was injected into the crude polymer solution and heated 150 °C for 10 min. After completion, excess amount of bromobenzene was injected into the reaction vessel and heated 150 °C for 10 min. The crude polymer was precipitated in methanol, collected by filtration, then loaded to an extraction thimble. The thimble was left into a soxhlet extractor, and the crude polymer was successively washed with methanol, acetone, and hexane. The resulting polymer was collected by chloroform. The chloroform solution with extracted polymer was concentrated and then precipitated into methanol. The precipitated polymer was collected by filtration and dried under high vacuum overnight, yielding the various studied NDI-based copolymers as dark blue solid.

Supplementary Table 2. Properties of DPP and NDI-based polymers. Number-average molecular weight and dispersity of molecular weight measured by high-temperature GPC eluent by trichlorobenzene at 180 °C.

Polymer	Acceptor unit	Donor unit (D1)	Donor unit (D2)	D1 : D2 feed ratio (molar ratio)	M_n (kDa)	M_w/M_n
DPP-0TVT	DPP	-	BT	0 : 10	38.5	2.56
DPP-2TVT	DPP	TVT	BT	2 : 8	44.4	2.98
DPP-5TVT	DPP	TVT	BT	5 : 5	34.9	2.91
DPP-8TVT	DPP	TVT	BT	8 : 2	43.1	2.57
DPP-10TVT (DPP-0TT)	DPP	TVT	-	10 : 0	34.5	2.35
DPP-10TT	DPP	-	TT	0 : 10	65.9	3.72
DPP-2TT	DPP	TVT	TT	8 : 2	52	2.4
NDI-0TVT	NDI	-	BT	0 : 10	48.2	5.99
NDI-8TVT	NDI	TVT	BT	8 : 2	40.8	2.08
NDI-10TVT	NDI	TVT	-	10 : 0	31	1.84

2. *Mechanical and electrical properties of prepared polymers*

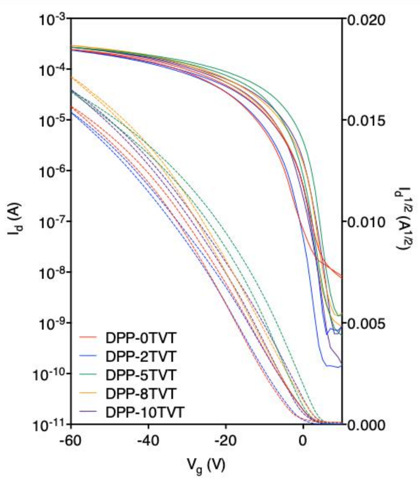

Supplementary Figure 3. Transfer curves of conjugated polymer films (DPP-0TVT ~ 10TVT) in air. The top-contact bottom-gate transistors described in Figure 1b were measured in air. Here, the drain voltage of -60 V was used. The channel length is 50 μm and the channel width is 1,000 μm.

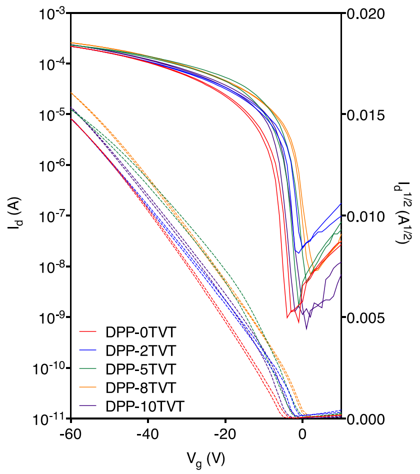

Supplementary Figure 4. Transfer curves of conjugated polymer films (DPP-0TVT ~ 10TVT) under nitrogen. The top-contact bottom-gate transistors described in Figure 1b were measured under nitrogen. Here, the drain voltage of -60 V was used. The channel length is 50 μm and the channel width is 1,000 μm.

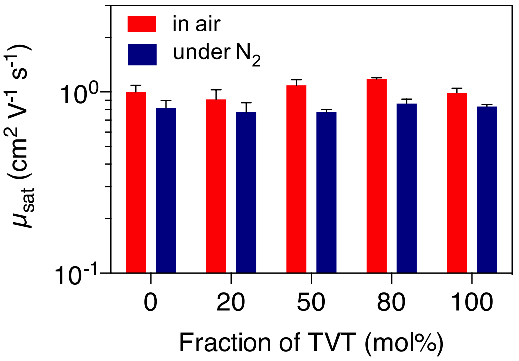

Supplementary Figure S5. Comparison of terpolymer mobilities measured in air (Figure 1b and Supplementary Figure S3) and under nitrogen (Supplementary Figure S4). The terpolymers showed slightly reduced mobilities under nitrogen. Error bars represent standard deviation.

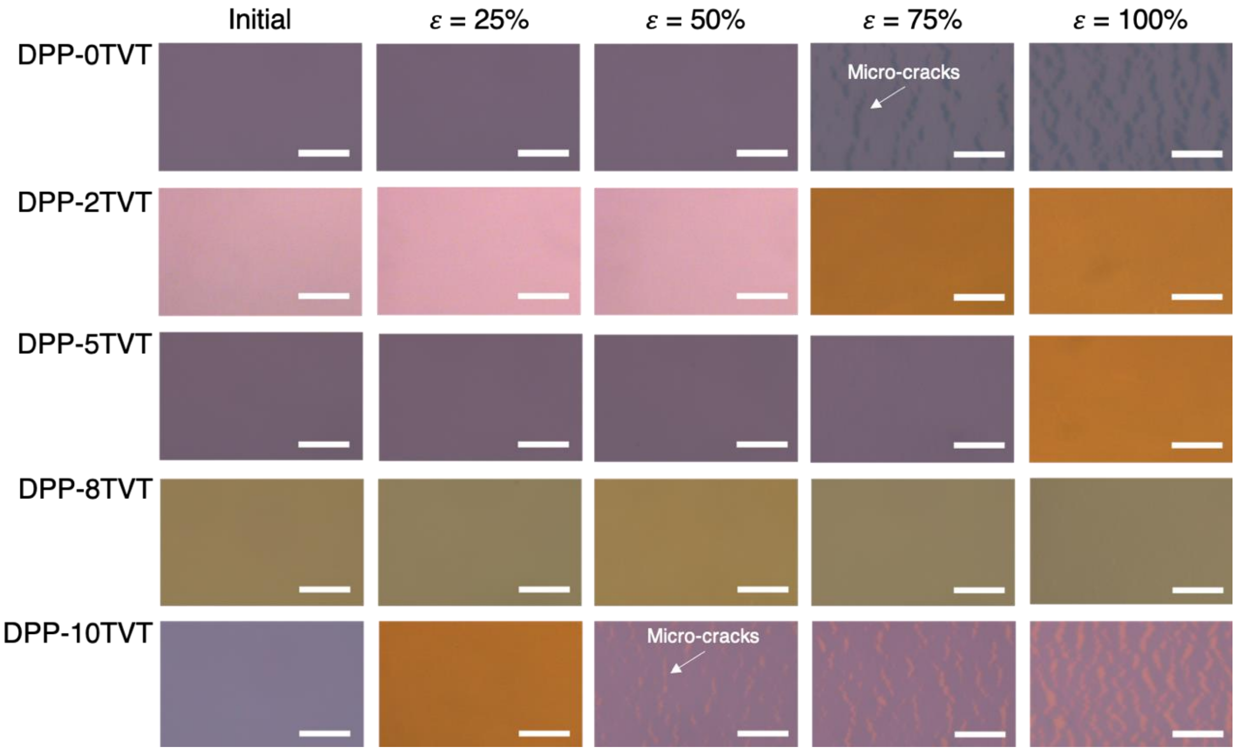

Supplementary Figure S6. Optical images of 30 nm thick semiconductor films at different strain levels. Terpolymer showed significantly higher strains before cracks are observed. (Scale bar: 10 μm)

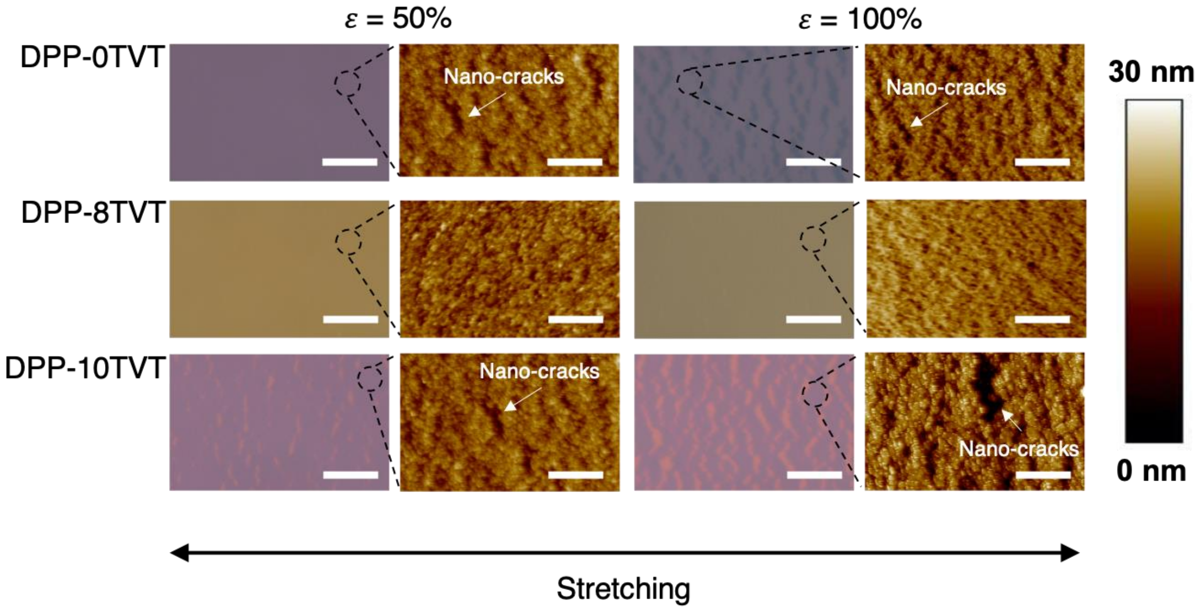

Supplementary Figure S7. Optical and tapping-mode AFM images of 30 nm thick semiconductor films at two different strain levels (50 and 100%). AFM was used to characterize thin films at smaller length scales. For AFM measurements, the areas that are visibly intact under an optical microscope were probed. The two reference polymers showed nano-crack formation and propagation even below their crack onset strain. However, no obvious cracks could be observed for DPP-8TVT, even at 100% strain. (Scale bar: 10 μm and 1 μm for optical and AFM images, respectively)

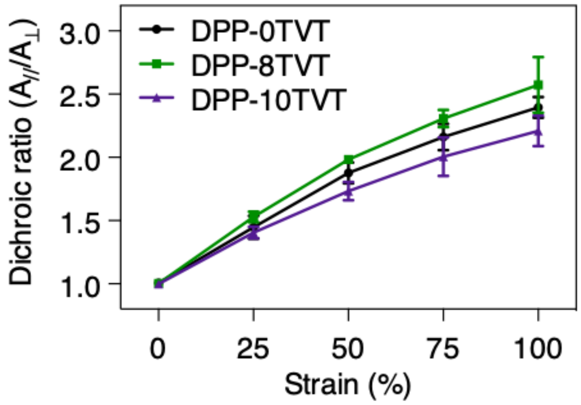

Supplementary Figure S8. The dichroic ratio of conjugated polymer films. DPP-8TVT showed larger dichroic ratio values than the two reference polymers at all strain levels. Since crack formation and propagation disrupts chain alignment under strain, these results suggest that DPP-8TVT showed the greatest stretchability. Error bars represent standard deviation.

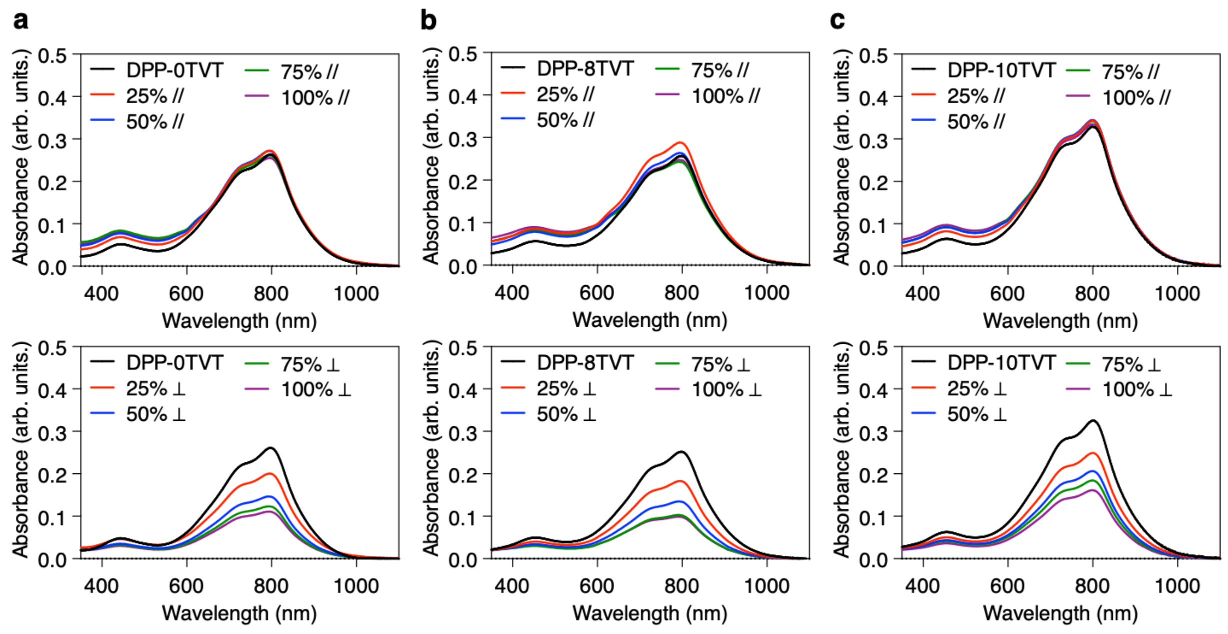

Supplementary Figure S9. UV-Vis-NIR absorption spectra of stretched films. UV-Vis-NIR absorption spectra of stretched (a) DPP-0TVT, (b) DPP-8TVT, and (c) DPP-10TVT films under a polarizer in the direction parallel and perpendicular to the strain. These spectra were used to calculate the dichroic ratio of the films under strain.

We showed that our terpolymers showed significantly improved crack onset strain without any compromise in mobility. To confirm that this improvement is independent of post-annealing of conjugated polymer films, we tested several annealing temperature and time. We chose DPP-8TVT for this testing because it showed the best mobility and stretchability. Note that molecular ordering may be improved with increasing annealing temperature and time, potentially decreasing stretchability. As shown in Supplementary Figure S10, it was clearly shown that DPP-8TVT exhibited high mobility (~ 1 cm^2 V^{-1} s^{-1}) with high crack onset strain greater than 100% strain. This result confirms that instead of post-annealing conditions, the molecular design of our terpolymers may be mainly responsible for high crack onset strain and high mobility.

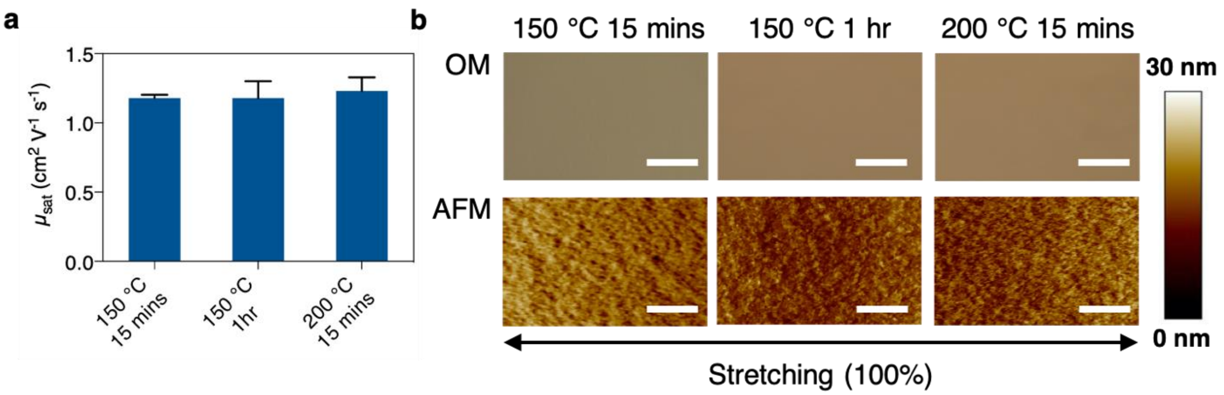

Supplementary Figure S10. DPP-8TVT films under several post-annealing conditions. (a) Mobility of DPP-8TVT annealed under different conditions. In all cases, DPP-8TVT showed high mobility near 1 cm^2 V^{-1} s^{-1}. Error bars represent standard deviation. (b) Optical microscope (OM) and AFM images of DPP-8TVT films under 100% strain. No crack was clearly observed in all images. The color scale of the AFM images represents relative height. (Scale bar: 10 μm and 1 μm for optical and AFM images, respectively)

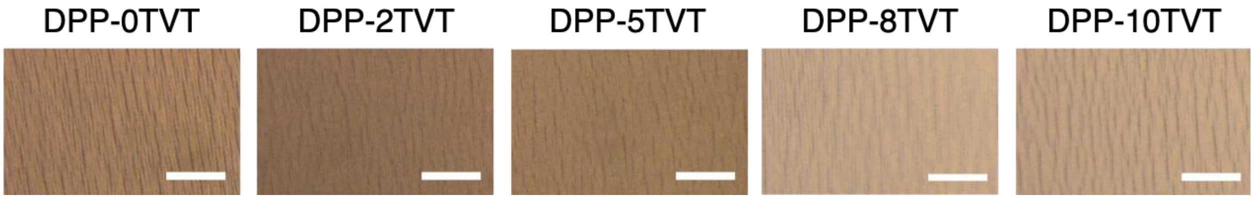

Supplementary Figure S11. Optical microscope images of 30 nm thick conjugated polymer films after the pre-strain of PDMS was released. The elastic modulus of the polymer films was determined from the wavelength of the wrinkles formed. (Scale bar: 10 μm)

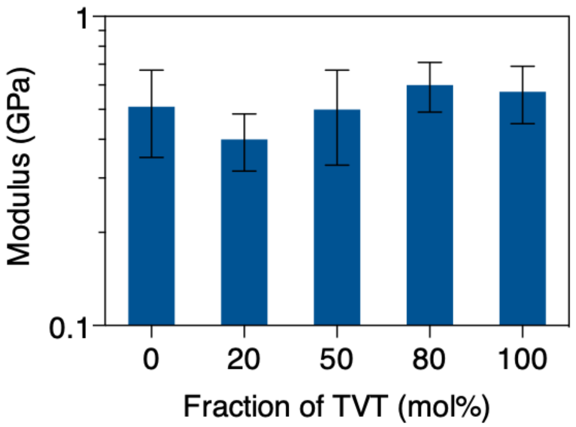

Supplementary Figure S12. The elastic modulus of 30 nm thick conjugated polymer films. Interestingly, the elastic modulus was unaffected by TVT fraction, probably because of fully conjugated backbones with similar chemical structures. We also reasoned that well-maintained overall ordering (characterized by UV-Vis-NIR spectroscopy) is responsible for unaffected modulus. Error bars represent standard deviation.

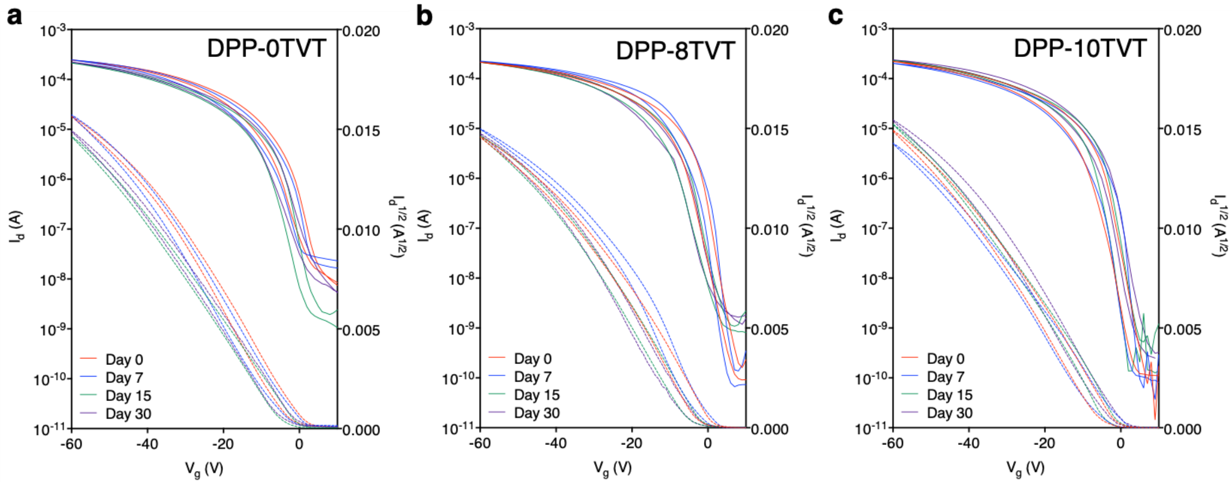

Supplementary Figure S13. Transfer curves of DPP-0TVT/8TVT/10TVT over time. All of the DPP-based semiconductors showed negligible change of transfer curves up to 30 days, which indicates that the terpolymers are air-stable.

In addition to stretchability and mobility, we also studied the mechanical reversibility of DPP-8TVT. For stretchable devices applications, stretchable semiconductors should be able to maintain their properties after repeated strain. Despite this importance, the reversibility of stretchable polymer semiconductors has rarely been studied. We tested DPP-0TVT, 10TVT (non-stretchable / high crystallinity), DPP-8TVT (stretchable / low crystallinity) and IDTBT (stretchable / amorphous). We showed that DPP-8TVT exhibited high stretchability and mechanical reversibility, while the other control samples compromised one of the two desired properties. Therefore, this result confirms that controlling multi-scale ordering from our terpolymer design effectively achieves high reversibility without significant compromise of stretchability.

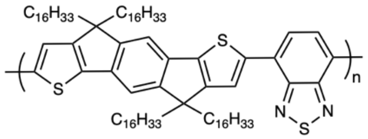

Supplementary Figure S14. Chemical structure of IDTBT. IDTBT was used as a control conjugated polymer.

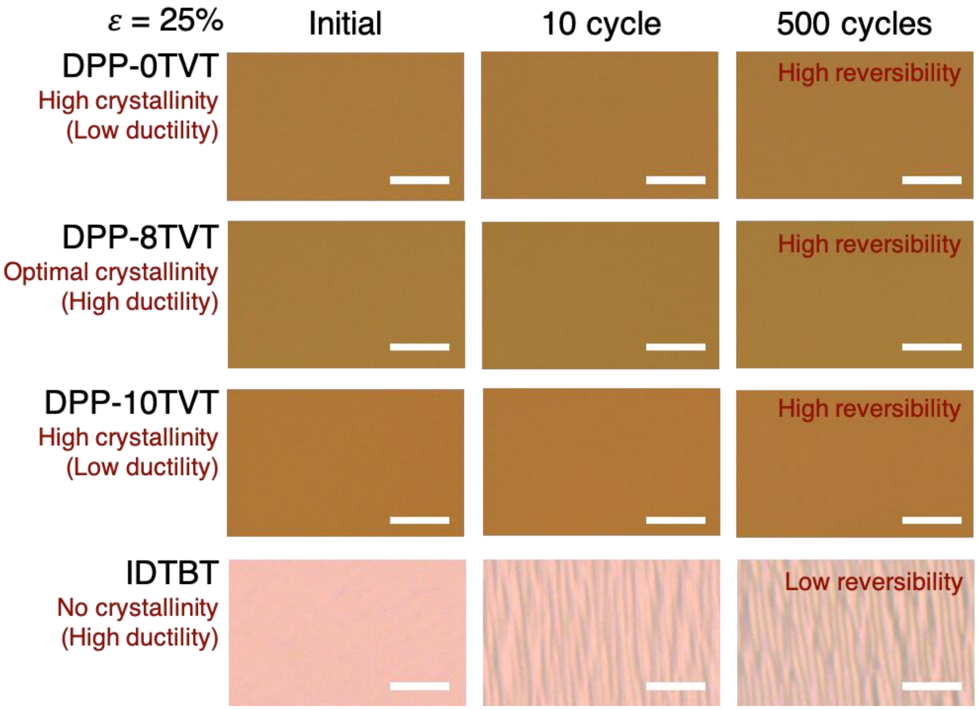

Supplementary Figure S15. Optical images of conjugated polymer thin films after repeated strain cycles between 0-25% strain. Semi-crystalline polymers (DPP-0TVT, 8TVT, and 10TVT) showed a negligible change, while the nearly-amorphous polymer (IDTBT) exhibited significant wrinkling, but no cracks. This result shows the importance of having crystalline domains in achieving reversibility. The crystalline domains are physical crosslinking sites. Combined with high crack onset strain data, DPP-8TVT showed optimal properties with both high stretchability and reversibility. (Scale bar: 10 μm)

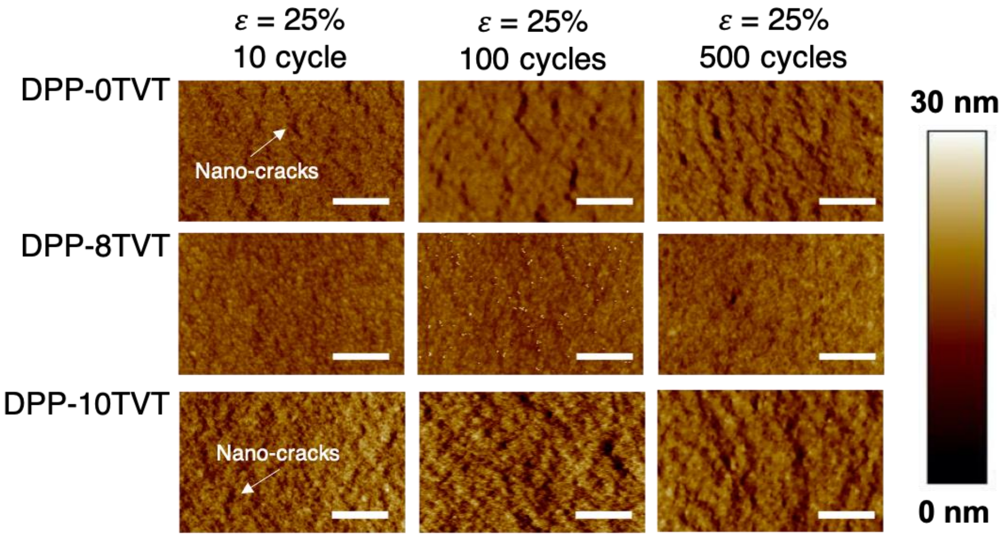

Supplementary Figure S16. Tapping mode AFM images of DPP-0TVT, 8TVT, and 10TVT after repeated strain cycles between 0-25% strain. At this smaller length scale, no wrinkling was observed. However, nano-cracks were observed in the higher crystalline films (DPP-0TVT and 10TVT). DPP-8TVT showed crack-free morphology without wrinkling. The color scale of the AFM images represents relative height. (Scale bar: 1 μm)

The above data indicated that DPP-8TVT has the most balanced stretchability and mechanical reversibility at 25% strain. We then tested the same properties under repeated 100% strain.

The optical images indicated that DPP-8TVT remained intact even under repeated 100% strain, while significant crack formation was observed for DPP-0TVT and 10TVT. (Supplementary Figure S17) This result indicates that DPP-8TVT has the highest stretchability, while the elastic limit of the three polymers may be above 100% strain in the micro-meter scale.

In the AFM images, DPP-0TVT and 10TVT exhibited significant micro-cracks and wrinkles. In contrast, DPP-8TVT showed significantly reduced crack formation. (Supplementary Figure S18) Again, this result confirms that DPP-8TVT showed significantly improved stretchability. The wrinkle formation under repeated 100% strain may indicate that the elastic limit of the terpolymers may be below 100% strain in the nano-meter scale. However, in our applications of stretchable transistors, the wrinkle formation in the nano-meter scale may be acceptable, since the devices are operated in the micro-meter scale.

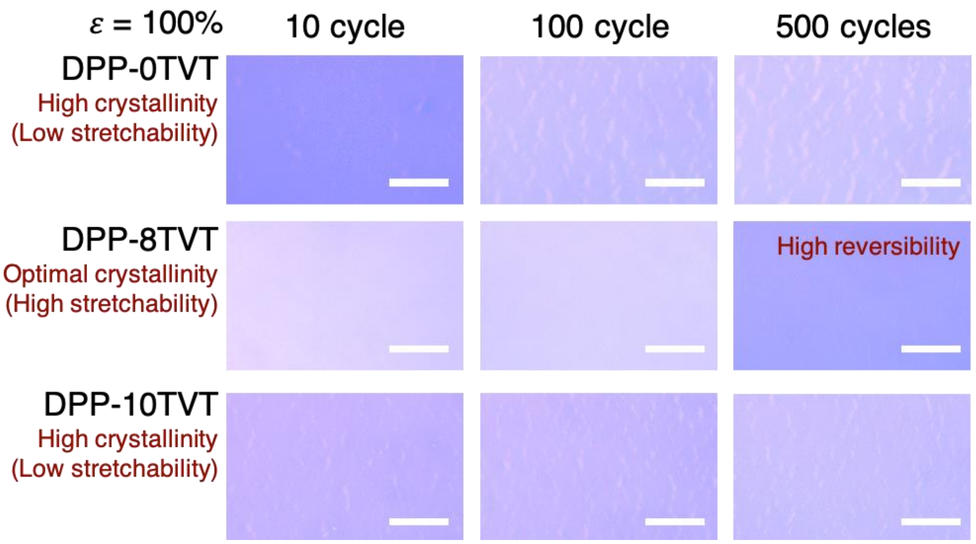

Supplementary Figure S17. Optical images of conjugated polymer thin films after repeated strain cycles between 0-100% strain. All of the semi-crystalline polymers (DPP-0TVT, 8TVT, and 10TVT) showed cracks at this high strain. (Scale bar: 10 μm)

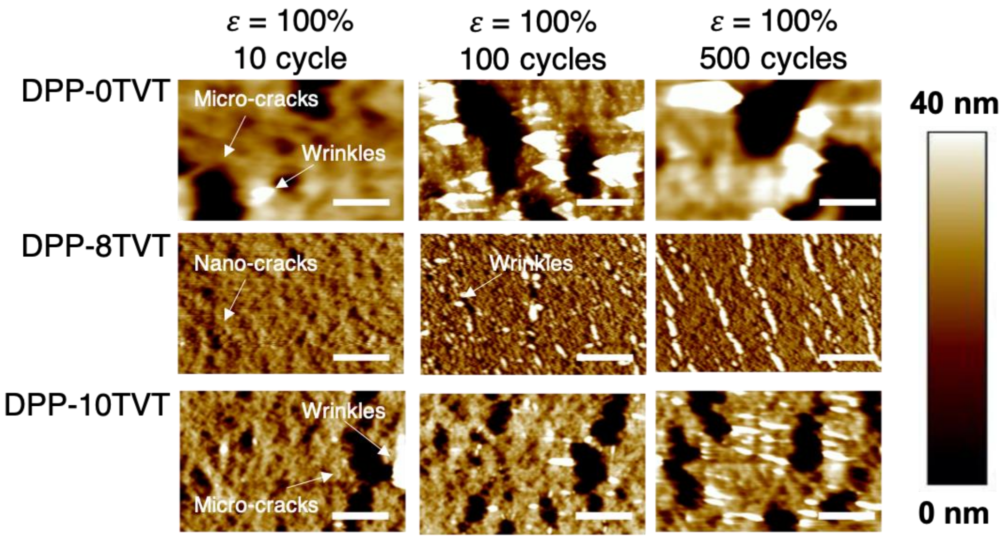

Supplementary Figure S18. Tapping mode AFM images of DPP-0TVT, 8TVT, and 10TVT after repeated strain cycles between 0-100% strain. Nano-cracks induced by repeated strains were observed in all of the films (DPP-0TVT, 8TVT and 10TVT). The color scale of the AFM images represents relative height. (Scale bar: 1 μm)

All of the above results showed how conjugated polymer films respond to repeated strain. Note that the high mechanical reversibility might be affected by molecular-level chain alignment and modulus of each polymer film.

To understand how polymer chains behave at the molecular level, we measured the dichroic ratio ($A_{//} / A_{\perp}$) of four polymers (DPP-0TVT, 8TVT, 10TVT, and IDTBT) after cyclic strain. This dichroic ratio characterizes whether polymer chains are aligned after cyclic strain. If the dichroic ratio is greater than 1, polymeric chains are aligned in the strain direction. As shown in Supplementary Figure S19, no significant alignment was observed after 1,000 times of 25% strain, although some macroscopic wrinkling existed for IDTBT. This result indicates that such macroscopic wrinkling formation is not due to the inability for aligned polymer chains to return to un-aligned conformation at this strain level.

Another possibility of wrinkle formation is from the modulus mismatch between the IDTBTT film and underlying elastomeric substrate. As seen in Supplementary Figure S12, all of the modulus values of our DPP-based semiconductors are around 0.5 GPa. The modulus of IDTBT was reported to be 0.745 and 2.5 GPa depending on the characterization methods.[1] From the buckling methodology we used in this study, the modulus of IDTBT was determined as 0.70 GPa. Since the modulus difference between PDMS substrates (0.6 MPa) and the polymer films (0.4 – 0.7 GPa) was significantly large, we expected that the difference in the modulus of DPP-based and IDTBT polymers might only have small effects on wrinkle formation.

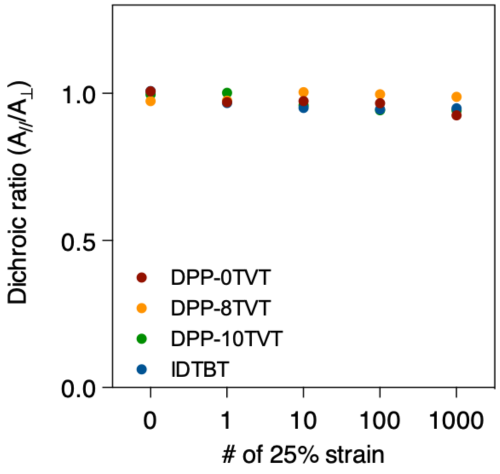

Supplementary Figure S19. Dichroic ratio of DPP-0TVT, 8TVT, 10TVT, and IDTBT after repeated strain. No significant chain alignment was observed even after 1,000 cycles of 25% strain.

3. Mechanisms behind the improved stretchability without compromise in mobility

With our terpolymer design, the common tradeoff between mobility and stretchability was completely avoided. In this section, the supplementary data are presented to explain such an improvement.

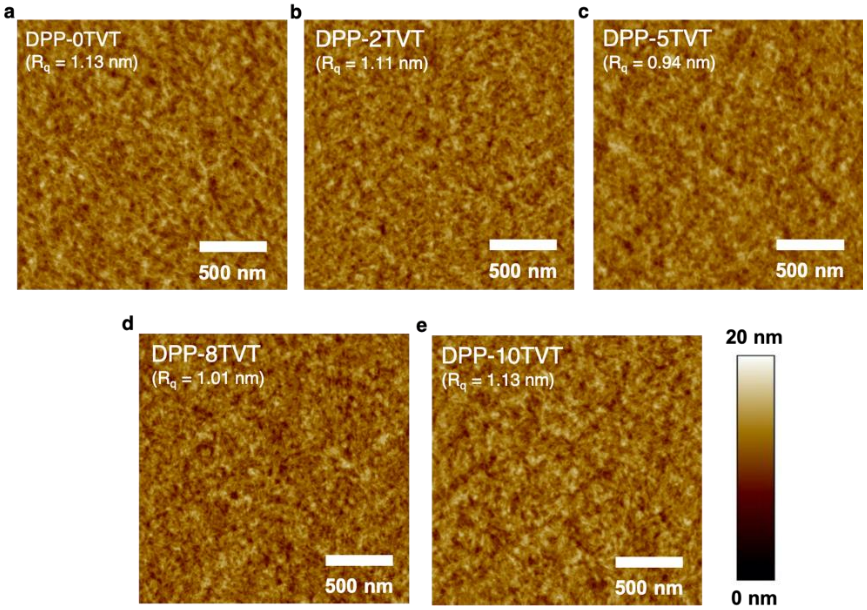

Supplementary Figure S20. Tapping mode AFM images of (a) DPP-0TVT, (b) 2TVT, (c) 5TVT, (d) 8TVT, and (e) 10TVT. All of the conjugated polymer films showed clear fiber-like morphology, which is desired for efficient charge transport. The color scale of the AFM images represents relative height.

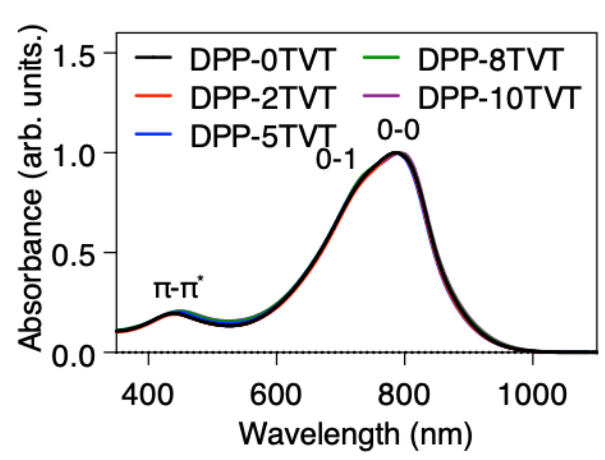

Supplementary Figure S21. UV-Vis-NIR absorption spectra of conjugated polymers with a concentration of 0.01 mg mL^{-1} in chlorobenzene. In both solution and thin film (Figure 2a) states, no difference in aggregation was observed between reference and terpolymer semiconductors. This well-maintained aggregation is responsible for efficient charge transport.

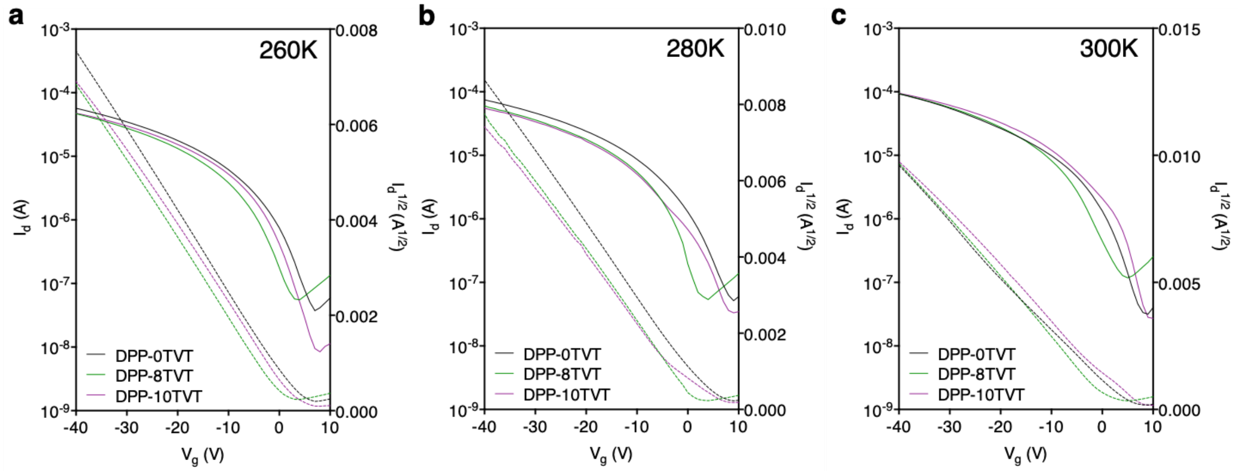

Supplementary Figure S22. Transfer curves of DPP-0TVT, 8TVT, and 10TVT at three different temperatures. The activation energy of charge transport was calculated from the mobility measured at these temperatures.

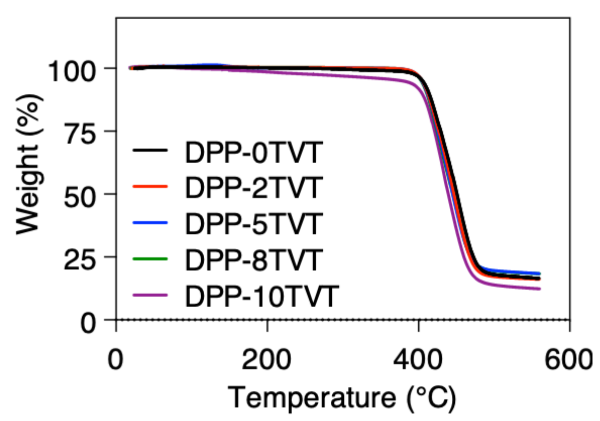

Supplementary Figure S23. TGA of conjugated polymers used in this study. The temperature scan range of DSC was determined from the decomposition temperature derived from TGA.

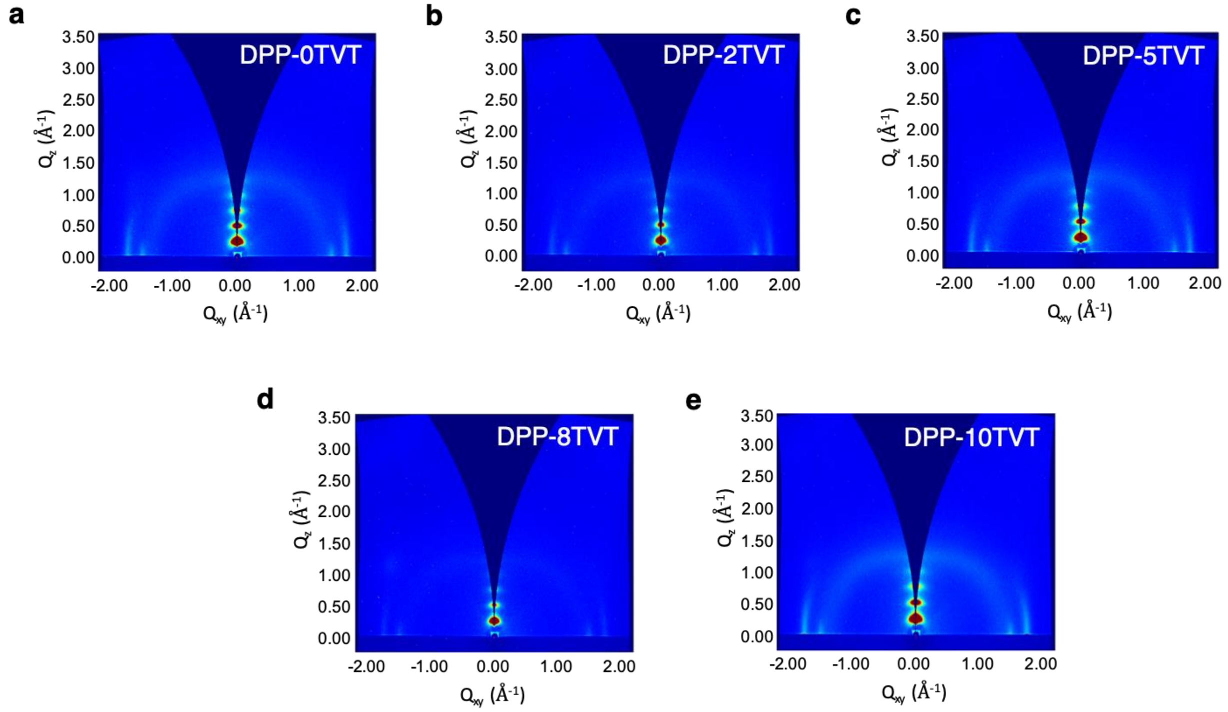

Supplementary Figure S24. GIXD images of conjugated polymers were used in this study. Compared with the two reference polymers (DPP-0TVT and 10TVT), our terpolymers (2TVT, 5TVT, and 8TVT) showed significantly reduced crystallinity, responsible for high stretchability. Still, the terpolymers exhibited clear semi-crystalline structures that enable high mechanical reversibility.

Supplementary Table S3. Detailed parameters of semiconducting polymer films obtained from GIXD analysis. Lamellar and π-π stacking distances show a decreasing trend with increasing TVT fraction. However, terpolymers (DPP-2TVT, 5TVT, and 8TVT) showed larger lamellar and π-π stacking FWHM and smaller coherence length than the other two reference polymers (DPP-0TVT and 10TVT). Coherence length was calculated from the (2 0 0) signal using the Scherrer equation. All of these results also indicate that the terpolymers have more disordered microstructures.

Polymers	Lamellar spacing (Å)	Lamellar peak FWHM (1/Å)	Coherence length (Å)	π-π spacing (Å)	π-π peak FWHM (1/Å)
**DPP-0TVT**	25.22	0.0600	94.2	3.59	0.102
**DPP-2TVT**	25.07	0.0629	89.9	3.58	0.114
**DPP-5TVT**	24.79	0.0622	90.9	3.56	0.104
**DPP-8TVT**	24.80	0.0650	87.0	3.56	0.107

**DPP-10TVT**	24.49	0.0613	92.4	3.55	0.102

The glass transition temperature (T_g) of the terpolymers may also have significant effects on the microstructures and the properties. However, the terpolymers used in this work were not sensitive enough to offer meaningful information of the glass transition in DSC and DMA. We expected that the difference in T_g may be negligible, given the structural similarity of the terpolymers. For example, the T_g values of DPP-0TVT and 10TVT with shorter side-chains (chemical structures are given in Supplementary Figure S25) were reported previously. Indeed, the difference in the T_g values was negligible.[2,3]

The T_g of the terpolymers can be derived from the Fox equation ($\frac{1}{T_g} = \frac{w_1}{T_{g1}} + \frac{w_2}{T_{g2}}$). From the Fox equation, the T_g values of the terpolymers may exist between those of the two reference polymers (DPP-0TVT and 10TVT). Therefore, we concluded that all of the terpolymers and the reference polymers may have similar T_g values, and the dynamicity of the polymeric chains may only have insignificant effects on the mechanical properties of the polymers.

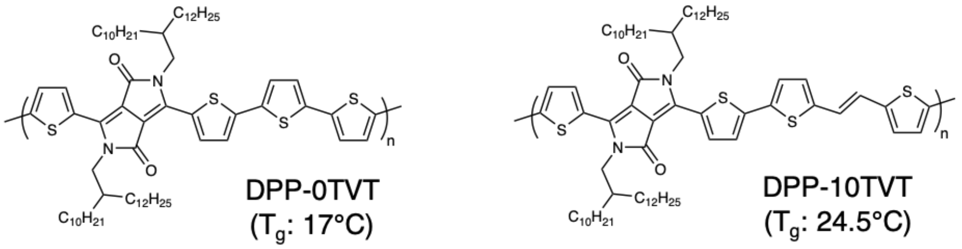

Supplementary Figure S25. T_g values of the DPP-0TVT and 10TVT reported in literature. Note that the reported polymers have shorter side-chains than the terpolymers used in this study.

4. Microstructure evolution of conjugated polymer films under strain

In order to understand the microstructure change of conjugated polymers, we performed GIXD measurements on stretched films. From the measurements, we could understand how each polymer dissipate applied mechanical stress. This section supplies supplementary data about microstructures of DPP-0TVT, 8TVT, and 10TVT under strain.

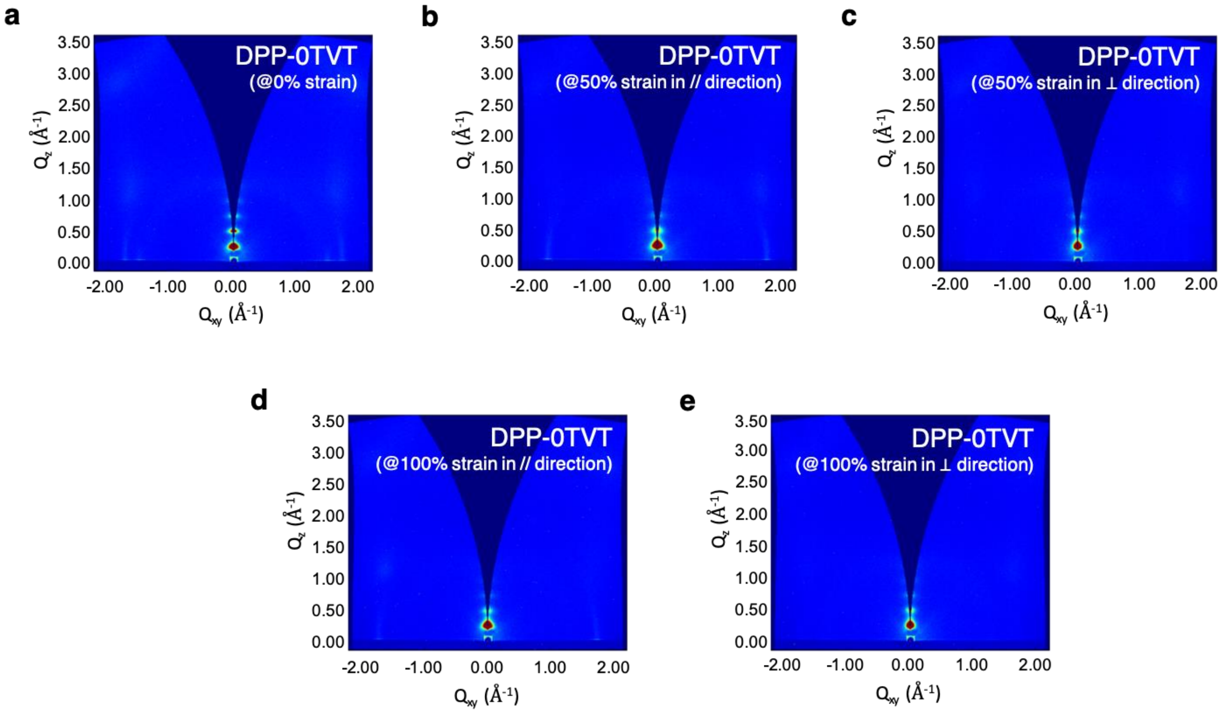

Supplementary Figure S26. GIXD images of DPP-0TVT stretched films. The microstructures of the stretched DPP-0TVT films were characterized at 0, 50, and 100% strain.

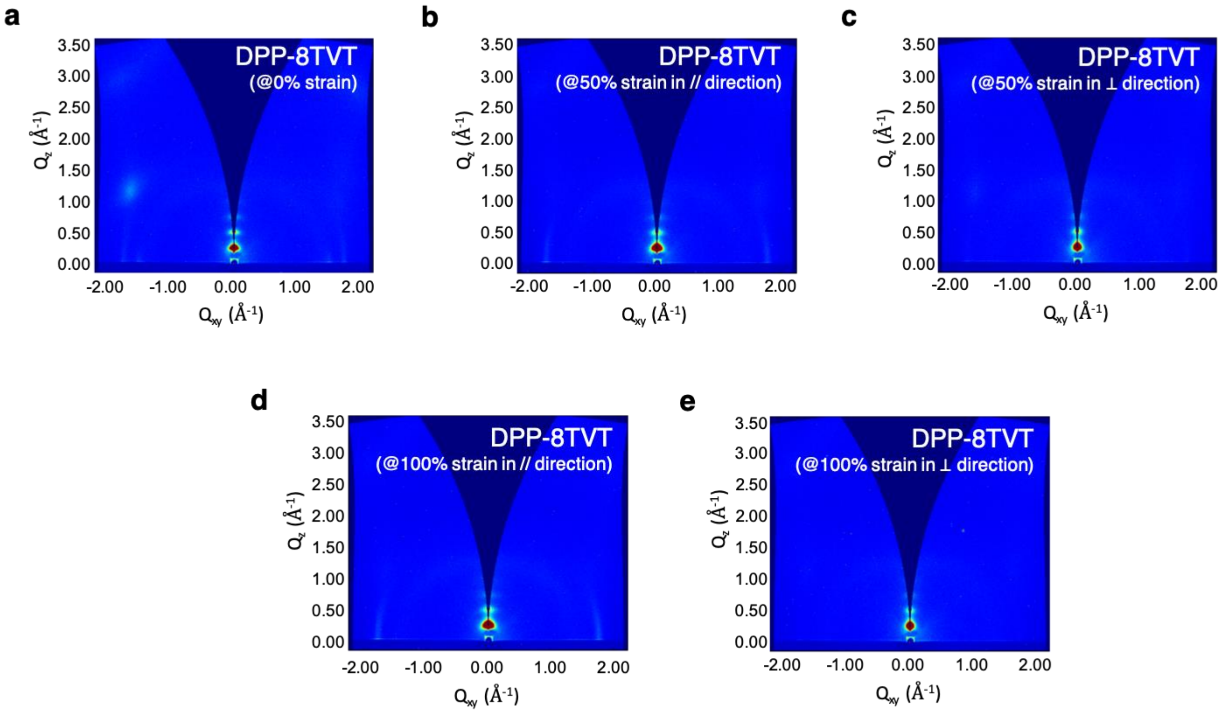

Supplementary Figure S27. GIXD images of DPP-8TVT stretched films. The microstructures of the stretched DPP-8TVT films were characterized at 0, 50, and 100% strain.

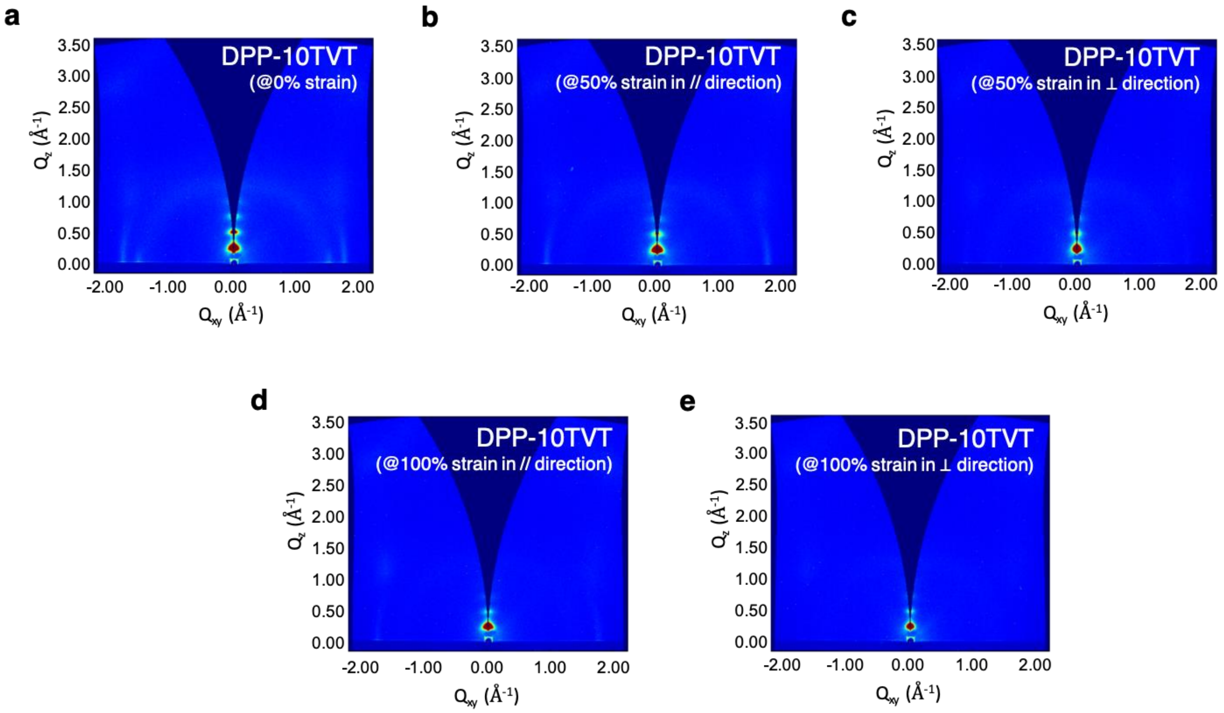

Supplementary Figure S28. GIXD images of DPP-10TVT stretched films. The microstructures of the stretched DPP-0TVT films were characterized at 0, 50, and 100% strain.

In addition to the two parameters (lamellar stacking distance, and rDoC from (2 0 0) peaks) described in the main text, we also studied the change of rDoC extracted from (0 1 0) peaks. As described in Supplementary Figure S29, DPP-8TVT showed greatest rDoC in both parallel and perpendicular directions to the strain, which indicates that its crystalline domains were well-retained under strain. This result again confirms that the crystalline domains of DPP-8TVT were least damaged, followed by DPP-0TVT and 10 TVT. Note that the increase in rDoC in the strain direction might arise from chain alignments effects as shown in Supplementary Figure S8.

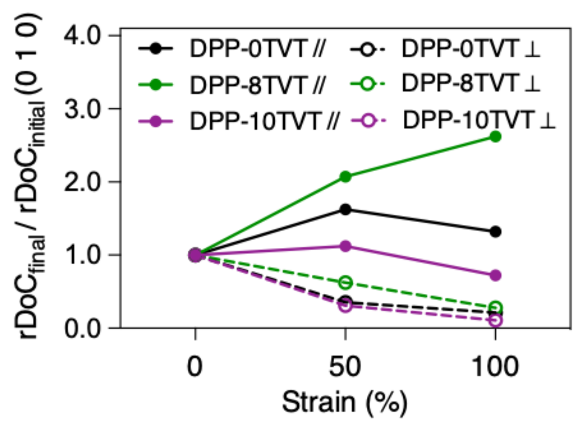

Supplementary Figure S29. rDoC change under strain extracted from GIXD. rDoC was calculated based on (0 1 0) peaks.

Supplementary Table S4. Detailed parameters of stretched semiconducting polymer films obtained from GIXD analysis. DPP-8TVT showed less significant microstructure change than DPP-0TVT and 10TVT, which indicates that crystalline domains of DPP-8TVT are less involved in the energy dissipation of the applied stress. Sufficient amorphous domains of DPP-8TVT effectively dissipated the stress. However, DPP-0TVT and 10TVT needed their crystalline domains to dissipate the stress since their amorphous domains are insufficient.

**Polymers**	**Strain**	**Lamellar spacing (Å)**	**Lamellar Peak FWHM (1/Å)**	***π-π* spacing (Å)**	***π-π* peak FWHM (1/Å)**
**DPP-0TVT**	0%	25.71	0.0515	3.60	0.093
**DPP-0TVT**	50% //	25.71	0.0716	3.58	0.104
**DPP-0TVT**	50% ⊥	25.69	0.0665	3.59	0.105
**DPP-0TVT**	100% //	26.10	0.0700	3.59	0.108
**DPP-0TVT**	100% ⊥	26.11	0.0653	3.59	0.102
**DPP-8TVT**	0%	25.10	0.0572	3.55	0.0899
**DPP-8TVT**	50% //	24.99	0.0711	3.55	0.1082
**DPP-8TVT**	50% ⊥	24.89	0.0678	3.55	0.0987
**DPP-8TVT**	100% //	25.13	0.0759	3.55	0.1076
**DPP-8TVT**	100% ⊥	25.17	0.0669	3.55	0.0876
**DPP-10TVT**	0%	24.75	0.0621	3.54	0.0941
**DPP-10TVT**	50% //	25.12	0.0733	3.56	0.1049
**DPP-10TVT**	50% ⊥	25.08	0.074	3.54	0.0949
**DPP-10TVT**	100% //	25.87	0.0687	3.55	0.1081
**DPP-10TVT**	100% ⊥	25.79	0.0672	3.54	0.1109

5. *Comparison of our terpolymers with blends*

We compared our terpolymers with blends of the two reference polymers. It was shown that terpolymers effectively reduced crystallinity without affecting aggregation, while polymer blends showed the negligible change of crystallinity and aggregation. This result may indicate that the two different polymer chains may phase-separate in the blends, while such phase separation is impossible in terpolymers because the two different monomer units are covalently linked.

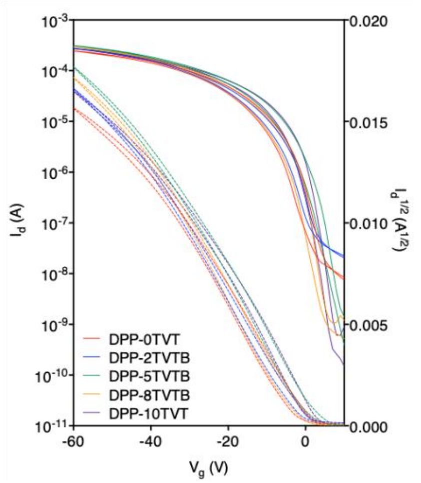

Supplementary Figure S30. Transfer curves of conjugated polymer blend films from top-contact bottom-gate transistors described in Figure 1b. Here, the drain voltage of -60 V was used. Note that all of the blend films showed negligible differences in charge transport.

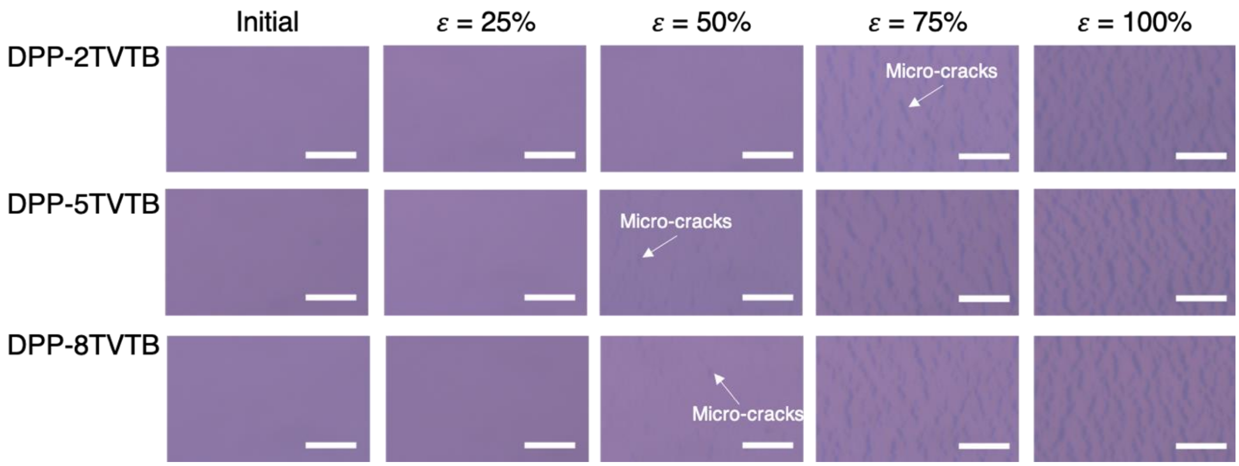

Supplementary Figure S31. Optical images of 30 nm thick semiconductor blend films at different strain levels. No significant improvement of stretchability was observed with polymer blends. (Scale bar: 10 µm)

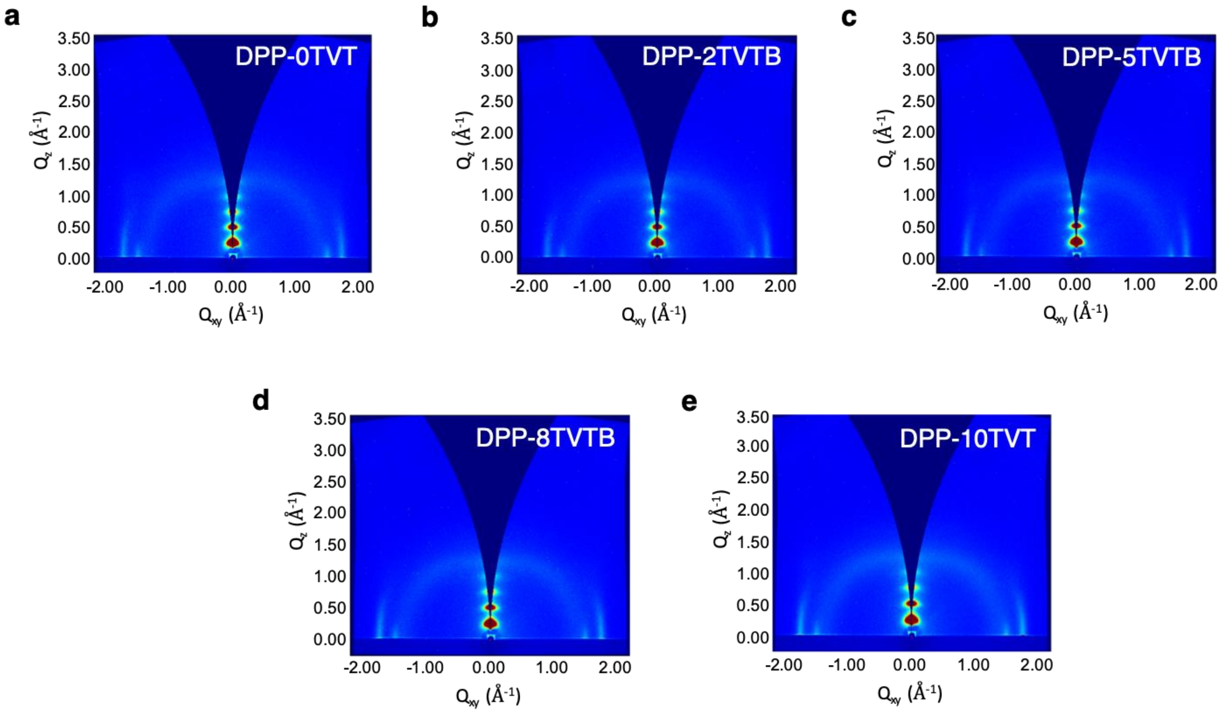

Supplementary Figure S32. GIXD images of blend semiconductor films. Compared with the two reference polymer films (DPP-0TVT and 10TVT), blend films (2TVTB, 5TVTB, and 8TVTB) showed an insignificant change of crystallinity, which is responsible for the negligible difference in stretchability.

Supplementary Table S5. Detailed parameters of semiconducting polymer blend films obtained from GIXD analysis. Lamellar and π-π stacking distances of the blend films showed a decreasing trend with increasing TVT fraction. Unlike terpolymers (DPP-2TVT, 5TVT, and 8TVT) reported in Supplementary Table S3, the blend films (DPP-2TVTB, 5TVTB, and 8TVTB) showed the negligible change of lamellar and π-π stacking FWHM compared with the two reference polymers (DPP-0TVT and 10TVT). This result indicates that the blend films have well-ordered microstructures, which might originate from phase separation of the two different polymer chains (DPP-0TVT and 10TVT).

**Polymers**	**Lamellar spacing (Å)**	**Lamellar Peak FWHM (1/Å)**	**π-π spacing (Å)**	**π-π peak FWHM (1/Å)**
**DPP-0TVT**	25.22	0.0600	3.59	0.102
**DPP-2TVTB**	25.09	0.0609	3.58	0.113
**DPP-5TVTB**	24.94	0.0586	3.58	0.096
**DPP-8TVTB**	24.69	0.0607	3.56	0.101
**DPP-10TVT**	24.49	0.0613	3.55	0.102

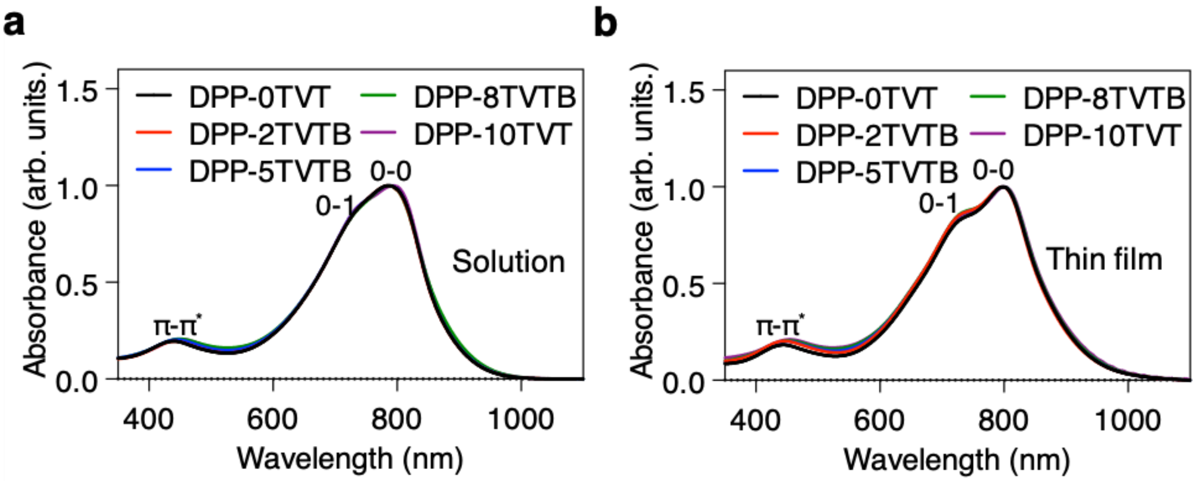

Supplementary Figure S33. UV-Vis-NIR absorption spectra of conjugated polymers of (a) 0.01 mg mL^{-1} solution in chlorobenzene and (b) 30 nm thick films. In both solution and thin film states, no difference in aggregation was observed between reference and blend semiconductors. This well-maintained aggregation is responsible for efficient charge transport.

6. *General applicability of our terpolymer design for developing stretchable polymer semiconductors*

In order to confirm that our design principle is generally applicable, we prepared two additional sets of polymer semiconductors with new combinations of donors and acceptors. We showed that our terpolymer design can significantly improve stretchability of conjugated polymers with little compromise in mobility.

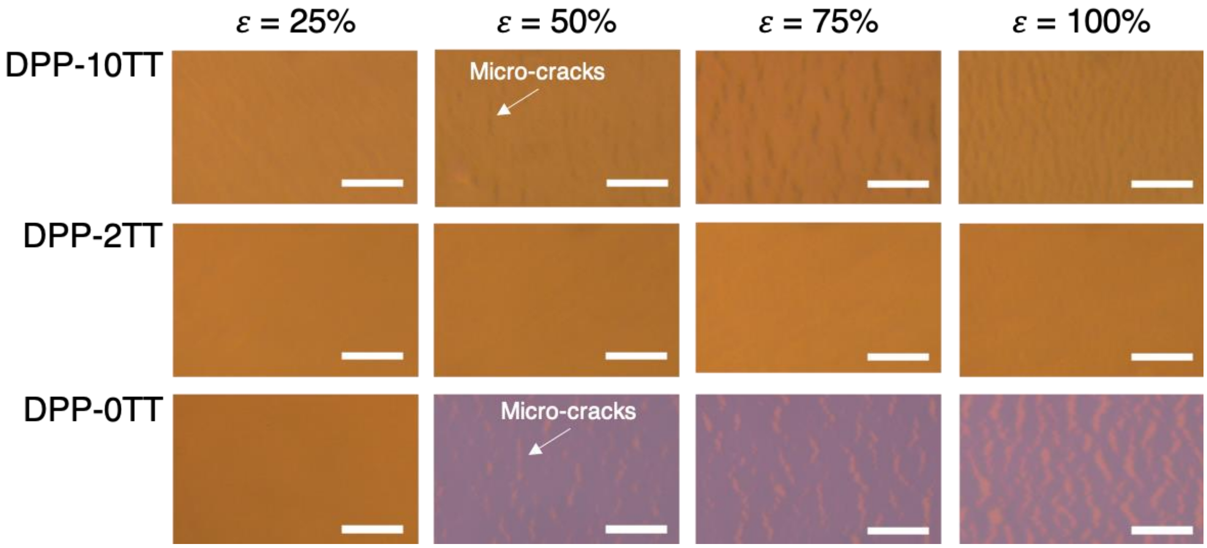

Supplementary Figure S34. Optical images of 30 nm thick semiconductor films at different strain levels. When TVT and TT were used as monomers, significant improvement of stretchability was again observed. (Scale bar: 10 μm)

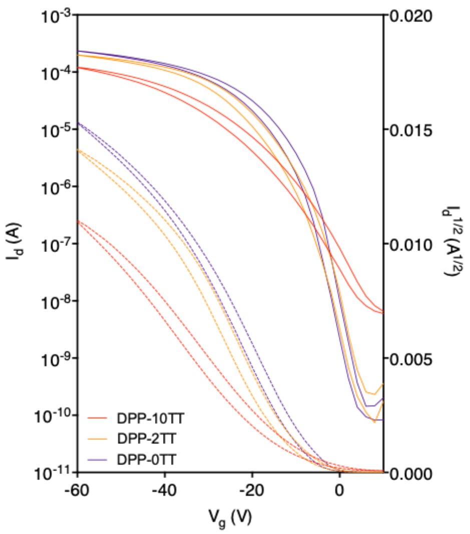

Supplementary Figure S35. Transfer curves of conjugated polymer films with TVT and TT as monomers. The top-contact bottom-gate transistors described in Figure 1b were measured in air. Here, the drain voltage of -60 V was used.

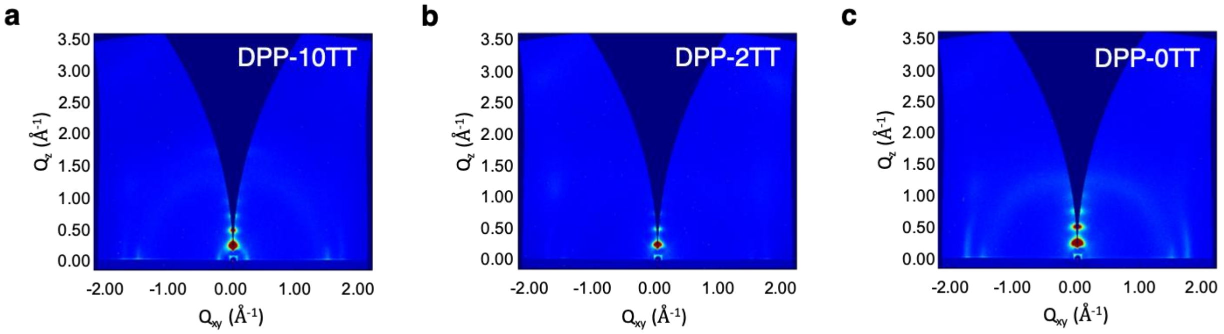

Supplementary Figure S36. GIXD images of conjugated polymer films with TVT and TT as monomers. Terpolymer films (DPP-2TT) showed significantly reduced crystallinity, which is responsible for a noticeable difference in stretchability.

Supplementary Table S6. Detailed parameters of semiconducting polymer films obtained from GIXD analysis. Although the relative degree of crystallinity of terpolymers (DPP-2TT) and NDI-8TVT) was significantly lower than the reference polymers, their FWHM values were similar or slightly lower, which allowed for efficient charge transport.

Polymers	Lamellar spacing (Å)	Lamellar Peak FWHM (1/Å)	π-π spacing (Å)	π-π peak FWHM (1/Å)
**DPP-10TT**	25.99	0.0647	3.58	0.114
**DPP-2TT**	25.47	0.0578	3.57	0.105
**DPP-0TT**	24.60	0.0627	3.55	0.098
**NDI-0TVT**	25.71	0.0685	3.83	0.0218
**NDI-8TVT**	26.30	0.055	3.79	0.0447
**NDI-10TVT**	26.21	0.0307	3.82	0.1808

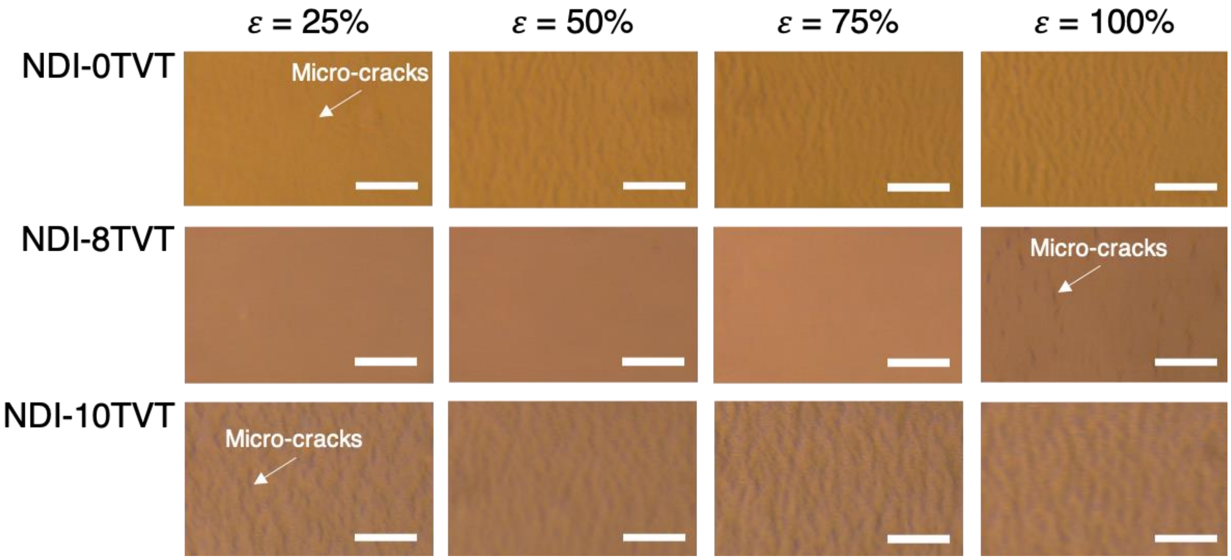

Supplementary Figure S37. Optical images of 30 nm thick semiconductor films at different strain levels. When NDI was used as a monomer, significant improvement of stretchability was

again observed. Note that the crack onset strain of NDI-8TVT was 30 times larger than that of NDI-10TVT. (Scale bar: 10 µm)

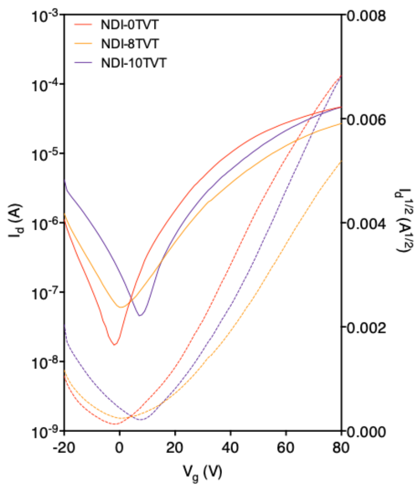

Supplementary Figure S38. Transfer curves of NDI-based polymer films under nitrogen. The top-contact bottom-gate transistors described in Figure 1b were measured under nitrogen. Here, the drain voltage of 80 V was used. Despite the 30-fold increase in stretchability, NDI-8TVT showed only a small decrease in mobility. The channel length is 50 µm and the channel width is 1,000 µm.

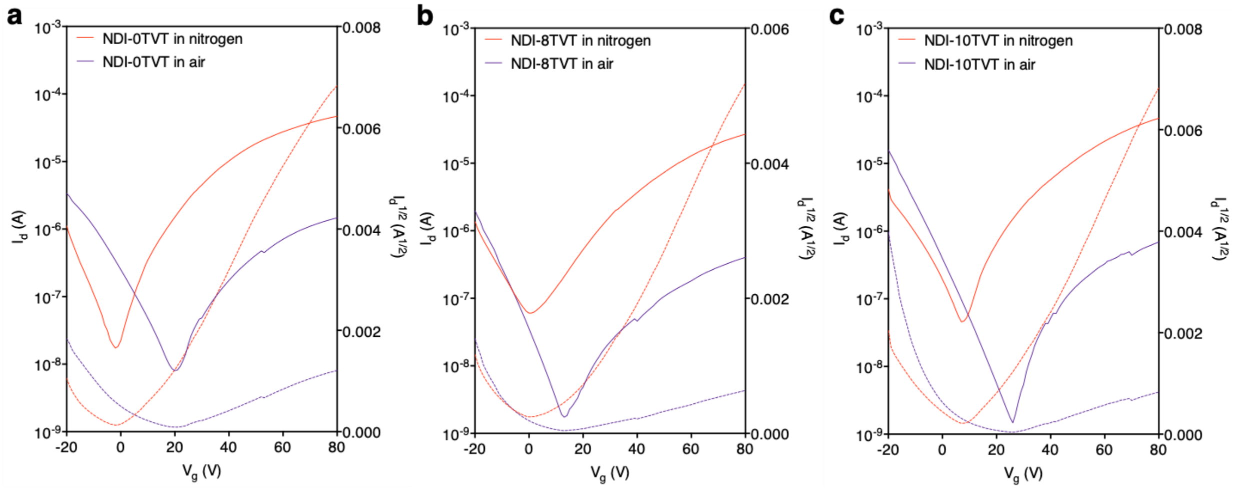

Supplementary Figure S39. Transfer curves of NDI-based polymer films in air. The top-contact bottom-gate transistors described in Figure 1b were measured in air. Here, the drain voltage of 80 V was used. The channel length is 50 µm and the channel width is 1,000 µm.

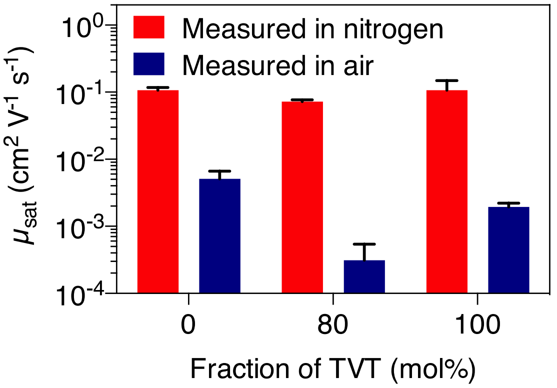

Supplementary Figure S40. Comparison of the mobilities of NDI-based semiconductors measured in air and under nitrogen. The NDI-based semiconductors showed a significant decrease in mobility in air, due to electron doping from oxygen/water molecules. These n-type semiconductors should be well-encapsulated in practical applications. Error bars represent standard deviation.

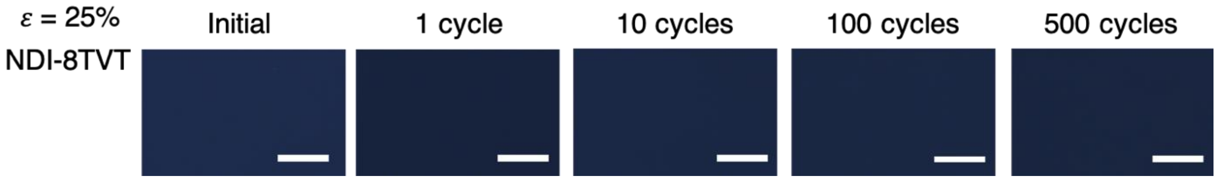

Supplementary Figure S41. Optical images of NDI-8TVT thin films after repeated strain. Instead of clear wrinkling, slight film non-uniformity was observed induced by cyclic strain. Combined with stretchability data, NDI-8TVT showed balanced stretchability and reversibility. (Scale bar: 10 μm)

7. Fully stretchable transistors and demonstrations

With our newly established stretchable polymer semiconductors, we fabricated fully stretchable transistors as a potential application. DPP-8TVT showed record-high mobility retention capabilities against single tensile and repeated strain.

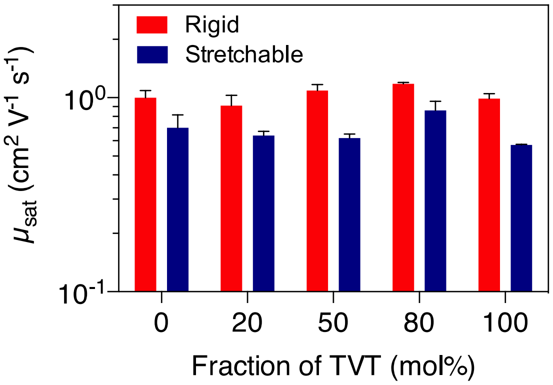

Supplementary Figure S42. Comparison of terpolymer mobilities measured from rigid (Figure 1b) and fully stretchable (Figure 5b) transistors. A slight decrease in mobilities was observed with stretchable transistors. This decrease might be due to the lack of octadecyltrimethoxysilane-treated substrates used in the rigid transistors, which is known to tune polymer semiconductors' microstructures. Error bars represent standard deviation.

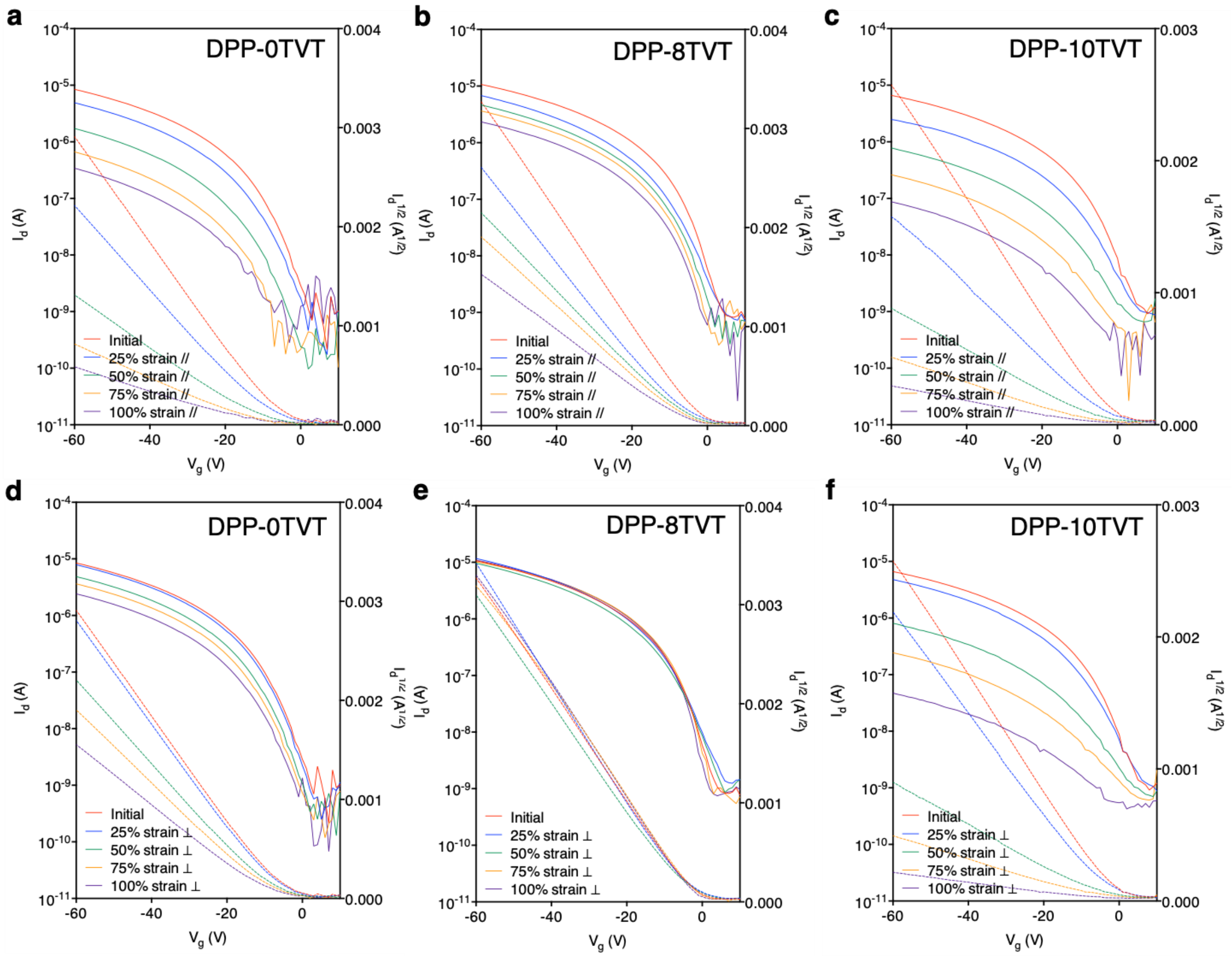

Supplementary Figure S43. Transfer curves of (a) DPP-0TVT, (b) 8TVT, and (c) 10TVT under strain measured from fully stretchable transistors. When compared with DPP-0TVT and 10TVT, DPP-8TVT exhibited significantly greater mobility retention.

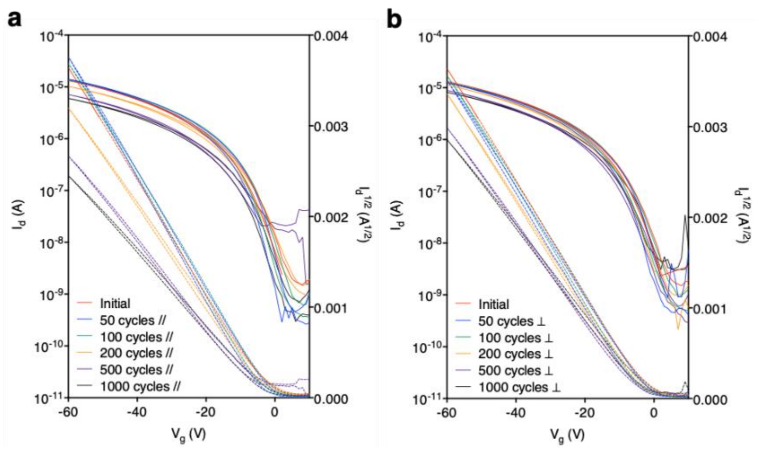

Supplementary Figure S44. Transfer curves of DPP-8TVT in the direction (a) parallel and (b) perpendicular to the repeated strain measured from fully stretchable transistors. Even after 1,000 times of 25% strain, DPP-8TVT maintained its mobility without significant change in threshold voltage.

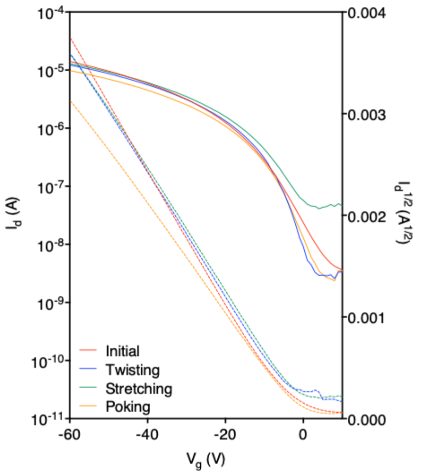

Supplementary Figure S45. Transfer curves of DPP-8TVT after various mechanical deformations. DPP-8TVT exhibited well-maintained mobility under twisting, stretching, and poking on a sharp object.

Supplementary Table S7. Comparison of our fully stretchable transistors with literature reported ones. Device structure, semiconductor material, and measurement conditions are summarized.

Device structure	Organic semiconducting materials	Measurement conditions	Reference
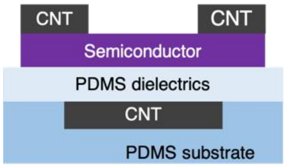	Terpolymer (DPP-8TVT)	In air	This work
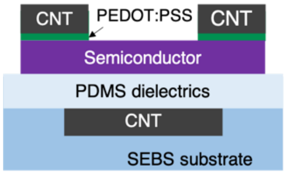	Energy dissipating semiconductor (DPP-10PDCA)	In air	32
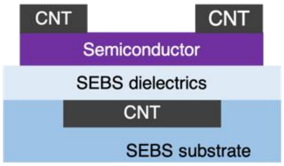	DPP-based semiconductor w/ conjugation breaker (C12-DPP)	In air	33
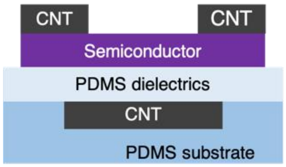	DPP-TT/F4-TCNQ	In air	35

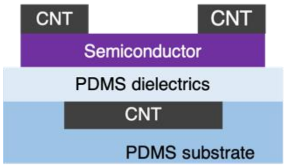	DPP-TT/CPP	In air	36
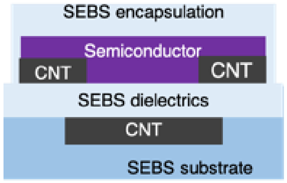	CONPHINE (PDPP-TT/SEBS)	In air	37
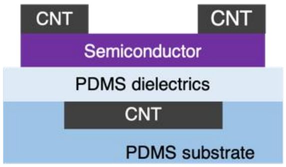	IDTBT	In air	41
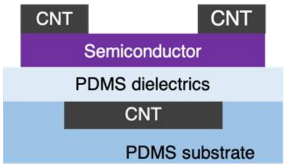	DPP-based semiconductor	In air	44
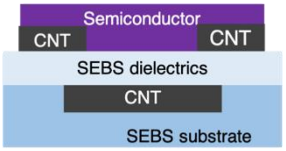	Solution-sheared CONPHINE	In air	45
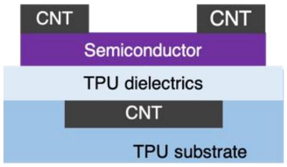	CNT	In air	51

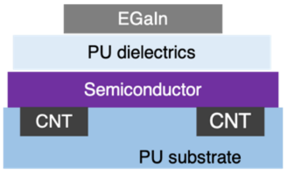	P3HT	In air	52

In order to show our terpolymer's suitability for device fabrication, we fabricated fully stretchable transistor arrays following the procedures reported previously.[4] As shown in the figures below, DPP-8TVT exhibited uniform mobilities. (Average mobility: 0.28 ± 0.03 $cm^2 V^{-1} s^{-1}$) This result indicates that our terpolymers may be used for various stretchable electronics applications. Note that a decrease in mobility was observed in the fully stretchable transistor arrays, which has been the common trend observed previously.[5] This might originate from the following factors. First, there may be a charge injection barrier between the patterned CNT electrodes and the patterned semiconductor. Second, there might exist solvent-induced damages during the fabrication processes. Finally, the transfer-printed semiconductor might have slightly reduced mobility.

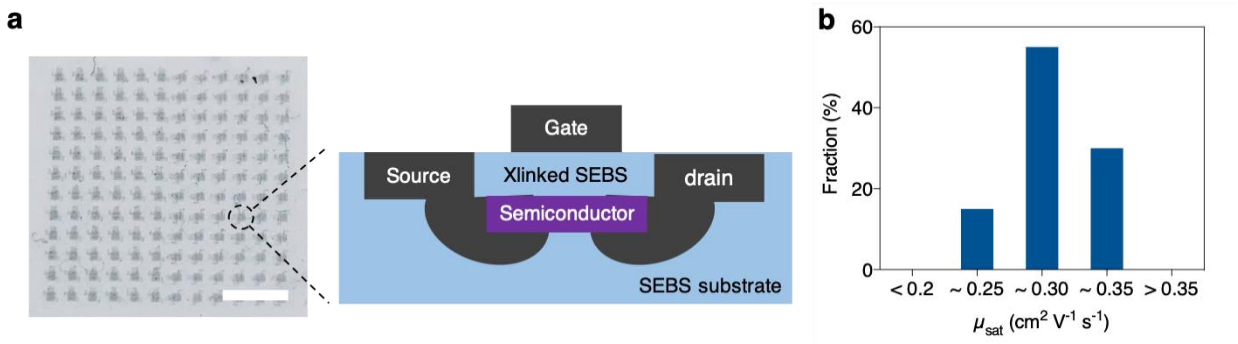

Supplementary Figure S46. Fully stretchable transistor arrays. (a) Picture and device scheme of 10 × 10 fully stretchable transistor arrays fabricated with DPP-8TVT. (Scale bar: 3 mm) CNTs were used for source, drain, and gate electrodes. DPP-8TVT was used as a semiconducting layer. Polystyrene-block-poly(ethylene-ran- butylene)-block-polystyrene (SEBS) was used for dielectrics and substrates. (b) Mobility distribution of the transistors.

References

1. Zheng, Y. *et al.* An Intrinsically Stretchable High-Performance Polymer Semiconductor with Low Crystallinity. *Adv. Funct. Mater.* **29**, 1905340 (2019).
2. Zhang, S. *et al.* The Critical Role of Electron-Donating Thiophene Groups on the Mechanical and Thermal Properties of Donor–Acceptor Semiconducting Polymers. *Adv. Electron. Mater.* **5**, 1800899 (2019).
3. Zhang, S. *et al.* Probing the Viscoelastic Property of Pseudo Free-Standing Conjugated Polymeric Thin Films. *Macromol. Rapid Commun.* **39**, 1800092 (2018).
4. Wang, S. *et al.* Skin electronics from scalable fabrication of an intrinsically stretchable transistor array. *Nature* **555**, 83–88 (2018).
5. Oh, J. Y. *et al.* Stretchable self-healable semiconducting polymer film for active-matrix strain-sensing array. *Sci. Adv.* **5**, eaav3097 (2019).
